# IR spectroscopic characterization of the co-adsorption of CO_2_ and H_2_ onto cationic Cu_*n*_^+^ clusters†

**DOI:** 10.1039/d1cp03119h

**Published:** 2021-10-28

**Authors:** Olga V. Lushchikova, Máté Szalay, Hossein Tahmasbi, Ludo B. F. Juurlink, Jörg Meyer, Tibor Höltzl, Joost M. Bakker

**Affiliations:** Radboud University, Institute for Molecules and Materials, FELIX Laboratory Toernooiveld 7 6525 ED Nijmegen The Netherlands joost.bakker@ru.nl; MTA-BME Computation Driven Chemistry Research Group, Department of Inorganic and Analytical Chemistry, Budapest University of Technology and Economics Muegyetem rkp. 3 Budapest 1111 Hungary; Leiden Institute of Chemistry, Gorlaeus Laboratories, Leiden University P. O. Box 9502 2300 RA Leiden The Netherlands; Furukawa Electric Institute of Technology Késmárk utca 28/A 1158 Budapest Hungary

## Abstract

To understand elementary reaction steps in the hydrogenation of CO_2_ over copper-based catalysts, we experimentally study the adsorption of CO_2_ and H_2_ onto cationic Cu_*n*_^+^ clusters. For this, we react Cu_*n*_^+^ clusters formed by laser ablation with a mixture of H_2_ and CO_2_ in a flow tube-type reaction channel and characterize the products formed by IR multiple-photon dissociation spectroscopy employing the IR free-electron laser FELICE. We analyze the spectra by comparing them to literature spectra of Cu_*n*_^+^ clusters reacted with H_2_ and with new spectra of Cu_*n*_^+^ clusters reacted with CO_2_. The latter indicate that CO_2_ is physisorbed in an end-on configuration when reacted with the clusters alone. Although the spectra for the co-adsorption products evidence H_2_ dissociation, no signs for CO_2_ activation or reduction are observed. This lack of reactivity for CO_2_ is rationalized by density functional theory calculations, which indicate that CO_2_ dissociation is hindered by a large reaction barrier. CO_2_ reduction to formate should energetically be possible, but the lack of formate observation is attributed to kinetic hindering.

## Abstract 2

To understand elementary reaction steps in the hydrogenation of CO_2_ over copper-based catalysts, we experimentally study the adsorption of CO_2_ and H_2_ onto cationic Cu_*n*_^+^ (*n* = 4–7) clusters.

## Graphical abstract

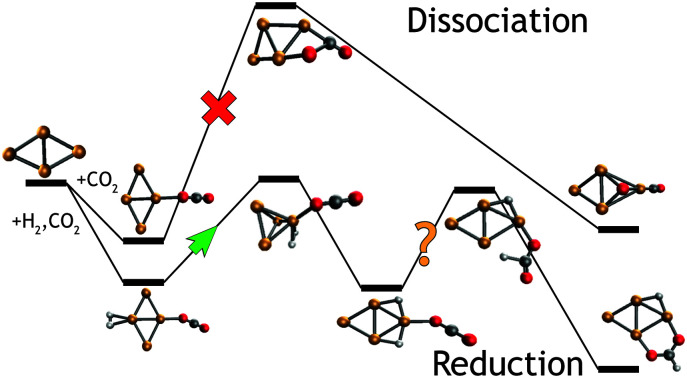

## Introduction

The rapid development of the urbanized world has resulted in the constant growth of the atmospheric CO_2_ concentration. On its own, CO_2_ is a harmless molecule, which is part of the natural carbon cycle. In large concentrations, CO_2_ acts as thermal isolation for our planet and is one of the main causes of global warming. Therefore, a reduction of the global carbon footprint is of great significance to reduce the societal impact of global warming.^1^

Since CO_2_ is non-toxic, renewable, and cheap, it has the potential to serve as a feedstock for the industrial production of value-added chemicals. The bottleneck of CO_2_ utilization is its kinetic inertness due to the high C=O bonding energy of 7.8 eV.^2^ Almost a century ago, syngas, a mixture of CO_2_, CO, and H_2_, was used for the first time for methanol production.^3^ This reaction required a catalyst, and elevated pressure (up to 350 bar) and temperature. Ever since, the search has been ongoing for more efficient catalyst materials that can reduce production costs, increase methanol selectivity, and minimize environmental impact.

The widely used Cu/ZnO/Al_2_O_3_ catalyst for commercial methanol production made it possible to reduce the reaction pressure to 50–100 bar at a temperature around 200–300 °C.^4^ Wide ranges of other catalysts have been investigated; a recent summary was given by Zhang *et al.*^5^ Overall, Cu based catalysts still show the best performance. However, the exact mechanism of methanol formation over the catalyst remains elusive. Theoretical studies have discussed several reaction paths.^6–8^ Some require the dissociation of hydrogen before CO_2_ activation, while others are initiated by direct CO_2_ activation or even water involvement. It is now widely accepted that the most profitable path for hydrogenation of CO_2_ to methanol over Cu-based catalysts proceeds *via* a formate (HCOO) intermediate.^4,6,9–12^

The surface chemistry of CO_2_ as studied under high vacuum conditions has been reviewed by various authors.^13–15^ Surface studies agree that the electronic and geometric structures determine the activity of the catalyst.^2,16–18^ Experimentally, it was shown that crystalline Cu(100) surfaces are relatively inert towards CO_2_.^16,19^ In the Cu/ZnO catalyst, Cu atoms are dispersed and Cu^+^ is stabilized,^20,21^ where a correlation was found between the concentration of Cu^+^ species on the catalyst surface and methanol production rates.^19,21–24^ Cu clusters deposited on a ZnO/Al_2_O_3_ surface were demonstrated to significantly reduce the pressure required for methanol formation.^25,26^ Concomitant density functional theory (DFT) modeling showed that the Cu clusters deposited on Al_2_O_3_ and SiO_2_ are slightly positively charged, which is attributed to the cluster–support interaction.^26^ Theoretical studies also emphasize that the size of the deposited cluster has a large influence on its activity, with smaller clusters showing a higher activity.^26–28^

Complete control over the structure and charge of the clusters is achieved in gas-phase studies. Here, metal clusters can mimic the catalyst active sites to study the elementary steps of methanol formation at the molecular level. Understanding the binding nature of CO_2_ to different metal ions has attracted broad research interest. The chemistry between cationic metal ions and a single CO_2_ molecule has been studied employing guided ion beam and flow tube reactor based mass spectrometry;^29–36^ a summary can be found elsewhere.^7^ IR spectroscopic studies have aimed to obtain structural insight into the solvent-driven activation of CO_2_ by various metal cations.^37–47^ The general picture that emerges is that metal cations mostly bind CO_2_ molecules intact, in end-on configuration *via* one of the oxygen atoms through quadrupole-related electrostatic interactions.

It can thus be expected that CO_2_ adsorption onto metal *clusters* is also dominated by a weak electrostatic interaction resulting in physisorption. However, what is unclear is how this situation changes when hydrogen atoms are present on the cluster surface. Hu *et al.* simulated methanol formation over a Cu_8_ cluster and compared it to the Cu(100) surface.^48^ According to this work, H_2_ and CO_2_ are co-adsorbed on the surface, where H_2_ is dissociated and CO_2_ is chemisorbed in the form of CO_2_^−^. This leads to a Langmuir–Hinshelwood type reaction between atomic hydrogen, H(a), and CO_2_^−^ resulting in a formate intermediate. Yang *et al.* also found that methanol formation will proceed *via* a formate intermediate, both over a Cu_29_ cluster, and over Cu(111).^49^ However, in this study the reaction proceeds *via* an Eley–Rideal-type mechanism. First, H_2_ dissociatively binds to the surface, and then CO_2_ directly reacts with the H(a) to form formate. Another mechanism of methanol formation over Cu/ZnO catalyst is proposed by Kakumoto.^11^ His calculations suggest that CO_2_ is first linearly absorbed on Cu^+^, followed by an attack of H(a) on the C atom, leading to the formate intermediate.^11^ Therefore, three alternative routes of the formation of the formate intermediate are proposed by the theoretical investigations.

Previously, we have shown that the adsorption of H_2_ onto cationic Cu_*n*_^+^ (*n* = 4–7) clusters can lead to a significant fraction of cluster population with dissociatively bound H_2_, with a size-dependent propensity for dissociation.^50^ In this work, we investigate the co-adsorption of CO_2_ and H_2_ on cationic Cu_*n*_^+^ clusters employing IRMPD spectroscopy. To interpret the co-adsorption spectra we compare them to equivalent spectra separately obtained for the adsorption products of the individual molecules. For H_2_ adsorbed on cationic Cu_*n*_^+^ we use spectra from our previous work;^50^ for CO_2_, we present new spectra. Furthermore, we have carried out extensive DFT calculations to rationalize and corroborate our interpretations.

## Methods

### Experimental

The experiments are performed using a molecular beam instrument placed within the cavity of a free-electron laser (FELICE).^51,52^ Cu clusters are produced in a Smalley–type laser ablation source, where a Cu rod is rotated and translated while being irradiated by the second harmonic (532 nm) of a pulsed Nd: YAG laser, focused on the rod to ablate metal atoms. For the spectroscopy of CO_2_ adsorption only, a rod of naturally abundant Cu (69% ^63^Cu and 31% ^65^Cu) is used. In the experiments utilizing both H_2_ and CO_2_, mass spectral overlap is prevented by employing a foil of isotopically enriched ^65^Cu (99.9%, STB Isotope GmbH) attached to a stainless steel rod. The plasma formed in the ablation process is collisionally cooled by a carrier gas pulse consisting of a mixture of 1% Ar in He for the CO_2_ experiment (to facilitate the formation of larger clusters) and of pure He for the co-adsorption experiment. The carrier gas is introduced *via* a pulsed valve (General Valve series 9) with a stagnation pressure of 6 bar. The created clusters are reacted with either pure CO_2_ or a mixture of 1% CO_2_ in H_2_, introduced approximately 50 mm downstream by a second pulsed valve with a 1 bar stagnation pressure. The reacting mixture is confined in the flow channel by a converging–diverging nozzle (diameter ∼0.7 mm), which is located 10 mm further downstream, through which the gas mixture eventually is expanded into vacuum, forming a molecular beam. The formed beam is subsequently collimated by a 2 mm diameter skimmer and shaped by a horizontal slit aperture (8 × 0.45 mm) to ensure optimum overlap with the (horizontal) IR beam, which it crosses at a 35° angle; both shaping elements are electrically grounded. The irradiated cationic species are extracted by two pulsed, high voltage plates into a reflectron time-of-flight mass spectrometer and registered by a multichannel plate (MCP) detector. The experiment is operated at 10 Hz, which is double the FELICE macropulse repetition rate. Therefore, for every mass spectrum of irradiated clusters recorded, a reference mass spectrum is recorded to correct for fluctuations in cluster production. When the IR light is resonant with an optically allowed vibrational mode of the complex, the sequential absorption of IR photons can lead to fragmentation, resulting in the loss of CO_2_, H_2_, or both.

The FELICE IR light employed in this work is in the 120–2100 cm^−1^ spectral range. Each FELICE macropulse is formed by a 10 μs duration pulse train of 1 ns spaced picosecond duration, near-transform limited optical pulses. The spectral bandwidth is adjusted to a full-width at half-maximum (FWHM) of about 0.7% of the central frequency, thus ∼7 cm^−1^ at 1000 cm^−1^. The typical macropulse energy used is between 0.5–0.8 J. The use of the intracavity instrument allows performing experiments, where high pulse energies are required for photofragmentation, as one could expect for complexes where CO_2_ and/or H_2_ adsorb dissociatively. For systems with lower binding energies, the instrument can be positioned well out of the focus of the IR laser to reduce the IR intensity. The IRMPD spectra presented in this paper are measured out of focus.^51^

The IRMPD spectra for CO_2_ adsorption are presented as the pulse energy-normalized depletion yield *Y*_D_, defined as
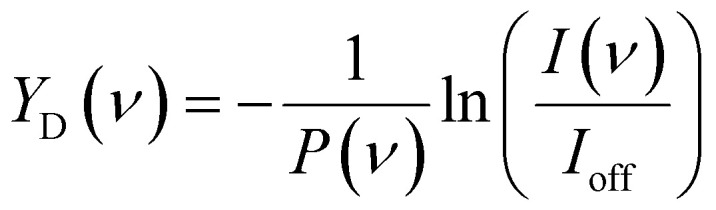
where *I*(*ν*) is the ion intensity of the mass channel under study with IR radiation at frequency *ν*, *I*_off_ the intensity without IR laser, and *P* the macropulse energy, respectively. If no fragmentation takes place, the ratio *I*(*ν*)*/I*_off_ is unity and *Y* is consequently zero.

**Scheme 1:**
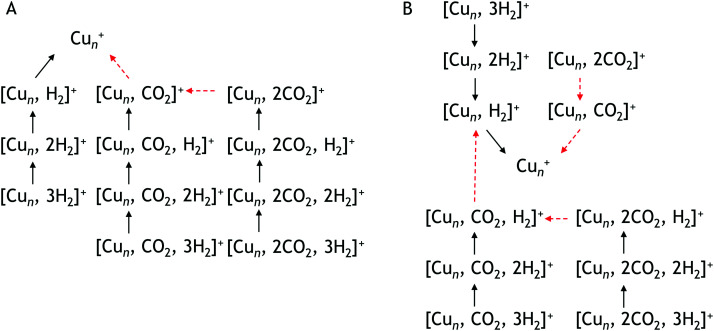
Observed fragmentation pathways. Pathway A is observed for Cu_4_^+^ pathway B for Cu_5_^+^, Cu_6_^+^, Cu_7_^+^. Black solid arrows indicate H_2_ loss, red dashed arrows CO_2_ loss.

To construct spectra for [Cu_*n*_, CO_2_, H_2_]^+^, loss of H_2_ from [Cu_*n*_, CO_2_, *p*H_2_]^+^ (*p* > 1) and of CO_2_ from [Cu_*n*_, *m*CO_2_, H_2_]^+^ (*m* > 1) leads to contamination of the mass channel of interest. To account for this, we inspected the IR-induced loss and growth signals for all relevant mass channels to identify the fragmentation pathways, which are summarized in Scheme 1; they are substantiated by depletion spectra per individual mass channel in the ESI.† We then calculate the branching ratio *B*(*ν*) of all clusters with *m* CO_2_ and *p* H_2_ molecules adsorbed to all these species plus the channel into which the [Cu_*n*_, CO_2_, H_2_]^+^ fragments. Because this channel is different for *n* = 4 (dominant loss channel [Cu_4_, CO_2_, H_2_]^+^ → [Cu_4_, CO_2_]^+^ + H_2_) than for *n* = 5–7 ([Cu_*n*_, CO_2_, H_2_]^+^ → [Cu_*n*_, H_2_]^+^ + CO_2_) the branching ratios are also different:
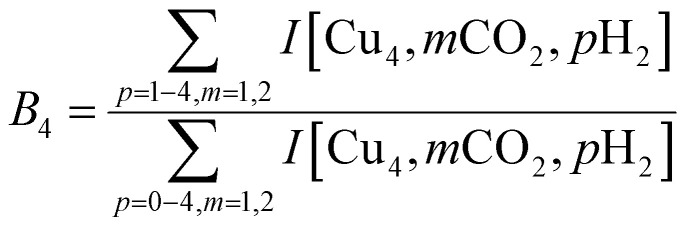

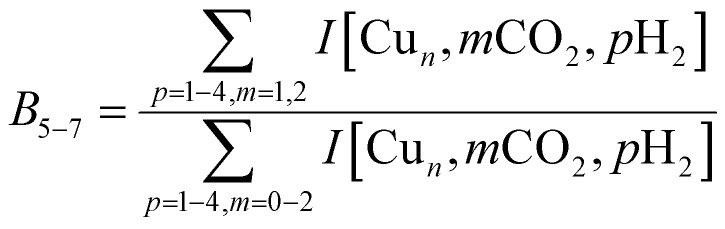


The IRMPD yield *Y*_B_ is then obtained as the logarithmic depletion ratio of the branching ratios with and without IR irradiation:
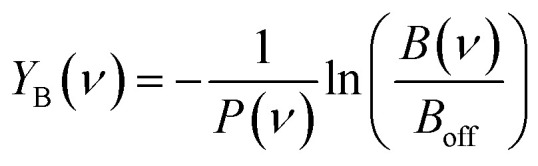


This approach assumes that no direct loss of multiple H_2_ or CO_2_ occurs from [Cu_*n*_, *m*CO_2_, *p*H_2_]^+^ (*m*,*p* > 1) induced by IR absorption, and was used previously.^53^ By employing it, ingrowth effects from higher order complexes are reduced, as are shot-to-shot fluctuations in cluster production.

To construct spectra for [Cu_*n*_, CO_2_]^+^ in the co-adsorption experiments, neither of the described methods is suitable. The depletion yield *Y* cannot be used since there is significant ingrowth from fragmentation from the different complexes into the [Cu_*n*_, CO_2_]^+^ mass channel. The depletion of the branching ratio also fails, because the final fragment in this case, the bare cluster, has competing ingrowth from fragmentation of [Cu_*n*_, *p*H_2_]^+^, which will contaminate the final spectrum. Therefore, the depletion yield *Y*_D_ of [Cu_*n*_, CO_2_]^+^ plus all species fragmenting into the [Cu_*n*_, CO_2_]^+^ mass channel has been calculated:
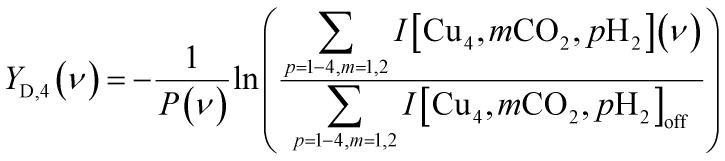

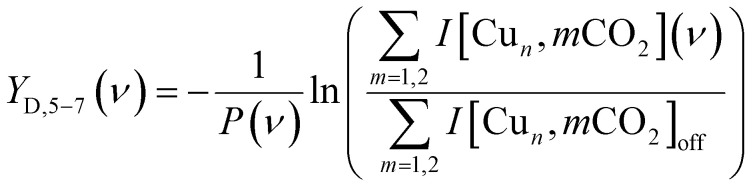


Throughout the manuscript, we employ the bracketed notation introduced here to indicate no structural knowledge of the products is known, only its mass.

### Computational

Quantum chemical computations were carried out using the Q-Chem 5.3 program package,^54^ and were mostly focused on the Cu_4_^+^ cluster. We performed optimizations on different initial cluster geometries (rhombohedral, tetrahedral, *etc.*) to locate the lowest energy bare Cu_4_^+^ cluster. Cluster geometries with different metal cluster cores and different CO_2_ and H_2_ binding motifs (*i.e.*, intact and dissociated H_2_ and CO_2_, CO_2_ bound with its oxygen or in di-σ binding mode) were systematically generated using our in-house code. This code, successfully used in other combined experimental and theoretical work on activation reactions involving metal clusters,^55–58^ systematically generates the initial cluster-adsorbate structures in different binding modes (see the ESI,† for the details). A publication detailing and benchmarking this software is forthcoming. The structures generated were optimized at the TPSSh/def2-TZVP + D3 level of theory.^59^ All calculations were done at the lowest spin multiplicity, *i.e.*, doublet for even and singlet for odd numbers of Cu atoms. The effect of the level of correlation for Cu_4_^+^ with CO_2_ or H_2_ adducts is compared to CCSD(T)/def2-QZVPPD benchmarks, while the finite basis size in DFT computations and relativistic effects were estimated using relativistic full-potential linearized augmented wave computations (see the ESI† for the details). We explored the reaction mechanisms between stable intermediates. Harmonic vibrational frequencies of the structures were computed for comparison with the experimental spectra, and to confirm that all minima and transition structures have zero or one imaginary vibrational frequency, respectively. The stability of the Self Consistent Field solution was confirmed. Intrinsic Reaction Coordinates were computed starting from the transition structures. To compare experimental spectra to calculated spectra, the harmonic frequencies were scaled by a factor 0.968 to correct for anharmonicity of the vibrational potential and inaccuracies of the employed level of theory. This factor was determined by fitting experimental band positions of [Cu_5_, H_2_]^+^,^50^ to calculated band positions of the assigned isomer at the current level of theory (see Fig. S4 in the ESI†). The scaled harmonic stick spectra were then convoluted with a 20 cm^−1^ full-width at half-maximum Gaussian line shape function. Enthalpies and Gibbs-free energies were computed at 298 K and 1 atm using the rigid rotor harmonic oscillator approximation and treating all internal degrees of freedoms as vibrations, *i.e.*, not considering hindered internal rotors. They are compiled in Table S1 in the ESI.†

## Results and discussion

### CO_2_ adsorption onto Cu_*n*_^+^ clusters

**Fig. 1:**
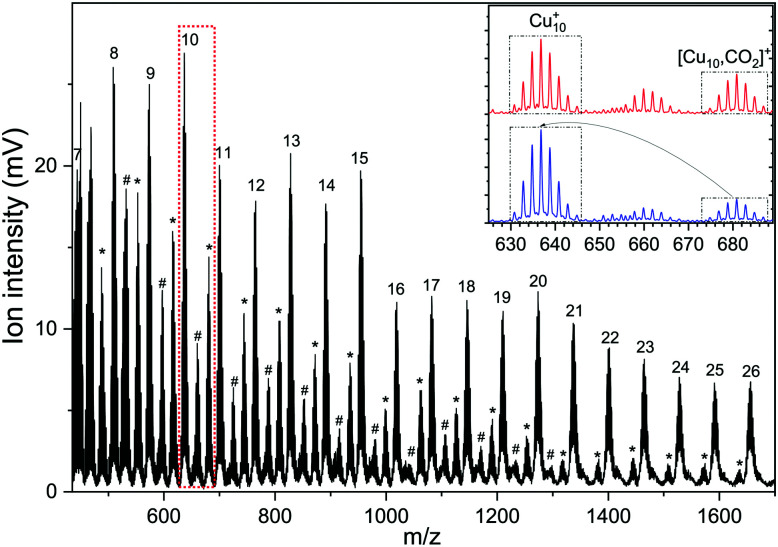
Time-of-flight mass spectrum of the [Cu_*n*_, *m*CO_2_]^+^ (*n* = 7–25, *m* = 0–2) complexes. Bare copper clusters are indicated by their number of constituent atoms n, complexes with *m* = 1 by a hash tag (#), with m = 2 with an asterisk (*). The inset shows a zoom-in displaying the isotopic distribution for Cu_10_^+^, [Cu_9_, 2CO_2_]^+^, and [Cu_10_,CO_2_]^+^, with IR light at 657 cm^−1^ (red trace) and without (blue trace), respectively.

A typical mass spectrum resulting from the reaction of cationic copper clusters Cu_*n*_^+^ (*n* = 7–25) with CO_2_ is shown in Fig. 1. We have found that production of Cu_*n*_^+^ (*n* = 1–4) in the presence of only a helium carrier gas yields significant signals for *n* ≤ 4; the production of larger Cu_*n*_^+^ clusters is facilitated by admixing a few % of Ar into the helium carrier gas. Since Ar readily binds to Cu_*n*_^+^ clusters, and especially so for the smaller clusters where the total charge is distributed over only a few atoms as was shown in our previous work,^50,60^ a competition between complexation of Cu_*n*_^+^ with Ar and CO_2_ results in a rather complex mass spectrum, with substantial mass overlap for the smaller cluster sizes. This overlap prevents us from completely disentangling depletion and growth for *n* < 7. In the mass spectrum shown, we observe that Cu_*n*_^+^ with 7 < *n* < 19 binds up to two CO_2_ with appreciable efficiency, and only one for *n* > 19. For the whole mass range, we observe no significant binding of Cu_*n*_^+^ with Ar, indicating that binding of CO_2_ is preferred here and that the binding energy thus likely exceeds the 0.2 eV found for Ar.^60^ The inset shows two mass spectra zoomed into the region of the Cu_10_^+^ cluster, where the isotopic distributions of Cu_10_^+^, [Cu_9_, 2CO_2_]^+^, and [Cu_10_, CO_2_]^+^ are visible. Upon resonant IR irradiation at a frequency of 657 cm^−1^, the distribution without IR light (top trace) changes such that the intensity of the [Cu_10_, CO_2_]^+^ distribution reduces, coinciding with an increase of the Cu_10_^+^ bare cluster distribution, as indicated with boxes and arrows in the bottom trace. This is indicative of photoinduced loss of CO_2_*via* the reaction [Cu_10_, CO_2_]^+^+ *k*·hν → Cu_10_^+^, with *k* an unknown number of IR photons.

**Fig. 2:**
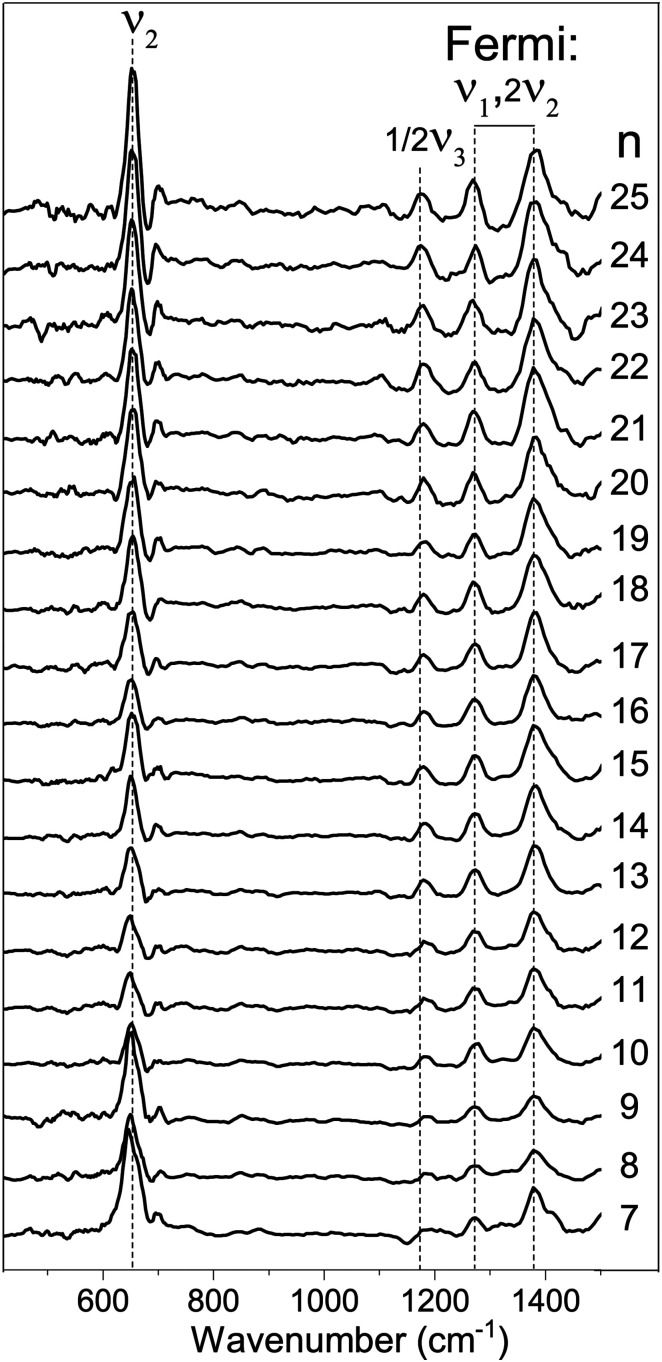
Experimental IRMPD spectra of [Cu_*n*_,CO_2_]^+^ (*n* = 7–25) in the 400–1500 cm^−1^ spectral range (the line a five-point adjacent-average). Dashed vertical lines are shown as guide to the eye.

In Fig. 2 the IRMPD spectra for [Cu_*n*_, CO_2_]^+^ (*n* = 7–25) are displayed. Whereas the adsorption of CO_2_ onto extended surfaces was shown either to lead to chemisorption or carbonate formation, depending on the surface morphology,^61^ clusters provide often very diverse structural motifs, and it can therefore *a priori* not be predicted whether the binding motif of CO_2_ will be similar for each cluster size. However, inspection of the spectra for different size *n* tells there are no significant differences. Although the signal-to-noise ratio gradually decreases with cluster size (a result of the decrease in production efficiency), it is evident that all spectra are very similar. We therefore discuss the spectrum of one cluster size in detail as a representative example for the other sizes. Cu_10_^+^ is chosen because a) the Cu_10_^+^ geometry is already known from our previous work on the spectroscopy of Cu_*n*_^+^·Ar clusters,^62^ and b) since a direct comparison between the spectrum of Cu_10_^+^·Ar and [Cu_10_, CO_2_]^+^ may allow to determine whether the adsorption of CO_2_ – be it dissociative adsorption, molecular chemisorption or physisorption – affects the structure of the cluster itself.

**Fig. 3:**
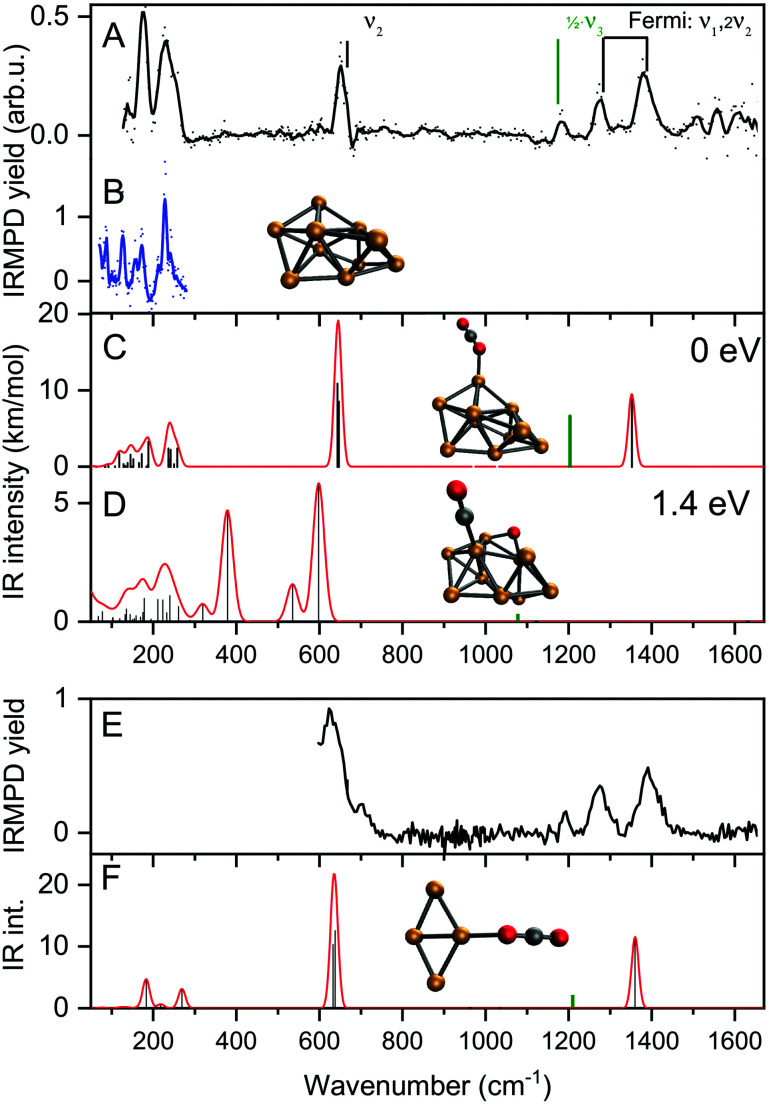
Experimental IRMPD spectra of [Cu_10_,CO_2_]^+^, [Cu_4_,CO_2_]^+^ (panels A/E, solid dots, the line a five-point adjacent-average) and Cu_10_^+^–Ar (panel B, in the metal–metal vibration range only). Panels C and D show the calculated vibrational spectra (red line) for two low-energy structures for complexes of Cu_10_^+^ with molecularly (panel C) and dissociatively absorbed (D) CO_2_, respectively. Panel F represents the calculated spectrum for [Cu_4_,CO_2_]^+^ with molecularly absorbed CO_2_. The green bar in panels C, D and F represents half the frequency of the CO_2_ asymmetric stretch vibration with the IR intensity divided by 100. The structures responsible for these spectra are shown with their relative energies of formation. The vibrational modes of free CO_2_ are indicated by vertical marks and their conventional mode labeling.

Fig. 3 shows the IRMPD spectrum of [Cu_10_, CO_2_]^+^ in the 120–1600 cm^−1^ spectral range (panel A) together with the IR photodissociation spectrum of Cu_10_^+^·Ar.^62^ Additionally, calculated spectra for two possible geometries, one with molecularly adsorbed CO_2_ (panel C) and one where one of the C=O bonds has been ruptured, and the eliminated O is separately adsorbed (panel D). The spectrum for [Cu_10_, CO_2_]^+^ exhibits seven clear bands with maxima at 134, 175, 231, 650, 1185, 1274, and 1378 cm^−1^, respectively, with a typical FWHM of 15–30 cm^−1^, with the exception of the band at 231 cm^−1^, for which the FWHM is about 50 cm^−1^. We attribute the oscillations in the spectrum above 1500 cm^−1^ to background noise that is amplified by normalization of the yield on the reduced IR laser pulse energies in this spectral area.

The typical vibrational modes of the Cu_10_^+^ cluster are in the 120–350 cm^−1^ region. Therefore, the first three bands (134, 175, and 231 cm^−1^) can readily be attributed to vibrations of the Cu_10_^+^ cluster. Comparison with the spectrum of Cu_10_^+^-Ar in Fig. 3B shows that the main bands are at the same line positions. The bands for [Cu_10_, CO_2_]^+^ are, however, significantly broader since the average FWHM of Cu_10_^+^–Ar is 11 cm^−1^.^62^ This band broadening could be caused by the higher binding energy of CO_2_ compared to that of Ar, but also by the larger pulse energies employed here. Nevertheless, the similarity between the spectra of the two complexes in the 120–350 cm^−1^ range strongly suggests that the cluster structure is not much affected by CO_2_ adsorption.

Before considering the current spectrum further, it is useful to discuss the vibrational structure of free CO_2_ in the gas phase. Free CO_2_ has three fundamental modes: the symmetric and anti-symmetric stretch vibrations *ν*_1_ and *ν*_3_, and the bending vibration *ν*_2_. Due to the symmetry of CO_2_, only *ν*_2_ and *ν*_3_ are IR-active, and they have been experimentally observed at 667 cm^−1^, and 2349 cm^−1^, respectively.^63^ The symmetric stretch is symmetry–forbidden in IR, but not in Raman spectroscopy. Here, not one but two bands are found, at 1388 cm^−1^ and 1285 cm^−1^, respectively. These bands are due to a Fermi resonance between *ν*_1_ and 2*ν*_2_.^63^

A rapid look at the experimental spectrum already indicates that the band at 650 cm^−1^ likely corresponds to the bending vibration of free CO_2_ (667 cm^−1^). Further, the series of three bands at 1185, 1274 and 1378 cm^−1^ are all in the region where the IR-forbidden symmetric vibrations are expected, which become IR active due to the cluster breaking the symmetry. However, for intact CO_2_ only two bands are expected, namely the two characteristic bands of the Fermi dyad. It is most likely that this dyad corresponds to the bands observed at 1274 and 1378 cm^−1^ for [Cu_10_, CO_2_]^+^.

Thus, only the band at 1185 cm^−1^ remains. This band cannot be a CO_2_ fundamental. It is also not an overtone of the bending vibration (667 cm^−1^ in free CO_2_) since this is already part of the Fermi dyad. Another possibility, a combination between the bending mode and a cluster vibration can be ruled out as none of the conceivable combination bands would have a frequency exceeding 1000 cm^−1^.

To seek a plausible explanation for the 1185 cm^−1^ band, we turn to DFT calculations. We did not do an extensive structure search, but rather limited ourselves to two possible structures, one with CO_2_ attached molecularly, and one where one of the CO bonds has ruptured, leaving a carbonyl and a separate O atom. In both cases, we assumed that the Cu_10_^+^ structure is the one found in experiments on Cu_10_^+^–Ar clusters.^62^ We first discuss the physisorbed species, for which the spectrum is shown in Fig. 3C. The binding energy of CO_2_ to Cu_10_^+^ in this species amounts to a mere 0.26 eV, which is only a little more than the Ar binding energy of 0.17 eV.^60^ Its calculated spectrum is dominated by a strong band at 645 cm^−1^, which is associated with the O=C=O bending motion. The bands at lower frequencies are the cluster vibrations, with little involvement of the CO_2_ ligand, or the vibrations involving the CO_2_–cluster bond (below 70 cm^−1^). The final bands are the modes associated with the CO_2_ symmetric (1352 cm^−1^) and anti-symmetric (2406 cm^−1^) stretching vibrations. Since this is a calculation in the harmonic approximation, a Fermi doublet caused by anharmonic couplings is not reproduced. The symmetric stretching mode at 1352 cm^−1^ is falling much closer to the higher two of the three bands in the 1100–1400 cm^−1^ spectral range, confirming that the 1274 and 1378 cm^−1^ experimental bands are indeed the Fermi dyad. Nevertheless, this species still does not provide a satisfactory explanation for the 1185 cm^−1^ band. Further trial structures of molecularly absorbed CO_2_ on Cu_10_^+^, all within 0.1 eV from the minimum shown here, have near-identical vibrational frequencies. An alternative structure conceivable is that of a dissociatively adsorbed CO_2_ where a separate O atom is bound to several Cu atoms, and a carbonyl CO is bound on an on-top site. While the on-top binding is typical for late transition metal surfaces, it is energetically quite unfavorable with a formation energy of 1.4 eV higher than the physisorption complex, and thus unlikely given its endothermicity. Indeed, the calculated spectrum does not give reason to suspect the species in the experiment is this structure, for instance an intense band at 378 cm^−1^ is not detected.

Thus, we find little reason to suspect the CO_2_ molecule is adsorbed in any other way than the physisorption complex shown in Fig. 3C. What is then the cause of the band at 1185 cm^−1^? For this, we have to critically evaluate the IR excitation laser and the possible presence of higher harmonics. It is well-known that any free-electron laser contains spontaneously emitted radiation at higher harmonic wavelengths, see *e.g.*,^64^ and lasing was achieved early on at the third harmonic.^65^ In contrast to odd harmonics, even harmonics have zero gain on the favored on-axis TEM_00_ optical mode. However, the less favorable TEM_01_ was demonstrated to have gain, and eventually lasing on the second harmonic was achieved at Jefferson Lab.^66^ Overall, one can thus expect that the second harmonic is present, albeit at substantially reduced intensity when compared to the harmonic. Inspection using a grating spectrometer indeed confirms that there is second harmonic radiation present with an intensity of ∼1% of the fundamental, making the observation of bands of similar oscillator strength as the ones discussed above unlikely. However, the one fundamental band not discussed as it is seemingly out of the spectral range probed, the CO_2_ anti-symmetric stretching vibration, has a much higher intensity than, *e.g.*, the bending mode (predicted 1203 and 17.4 km mol^−1^ for the physisorbed complex shown in Fig. 3C, respectively), and can thus plausibly be observed if the intensity of the second harmonic radiation is no less than three orders of magnitude lower. Thus, we assign the observed band at 1185 cm^−1^ to the anti-symmetric CO_2_ stretching mode, which thus has its band maximum at 2370 cm^−1^. This is slightly lower than the 2406 cm^−1^ predicted for the physisorbed Cu_10_^+^–CO_2_ complex, shown at half its frequency and 1/100 its calculated IR intensity by the green trace in Fig. 3C, but still altogether acceptable. Unfortunately, we are unable to verify this assignment experimentally with the FEL fundamental, since this is out of the spectral range covered by FELICE. Nevertheless, we can unambiguously conclude that the Cu_10_^+^–CO_2_ reaction product adopts a physisorption complex. Given the similarity of all spectra shown in Fig. 2, we conclude that the intermediate size cationic Cu_*n*_^+^ clusters all weakly bind CO_2_ and no significant activation takes place.

Panel E shows the spectrum recorded for [Cu_4_, CO_2_]^+^ using pure helium as carrier gas over the 600–1600 cm^−1^ spectral range. It essentially shows the same four bands as observed for [Cu_10_, CO_2_]^+^. The band at 630 cm^−1^ is somewhat broader and shows a high-frequency shoulder. The calculated IR spectrum for the Cu_4_^+^·CO_2_ physisorption complex shows the two CO_2_ bending and symmetric stretch vibrations at near-identical frequencies as for Cu_10_^+^·CO_2_ (panel C). In summary, like the larger Cu_*n*_^+^ clusters, CO_2_ adsorbs molecularly on Cu_4_^+^.

### Co-adsorption of H_2_ and CO_2_ onto Cu_*n*_^+^ clusters

**Fig. 4:**
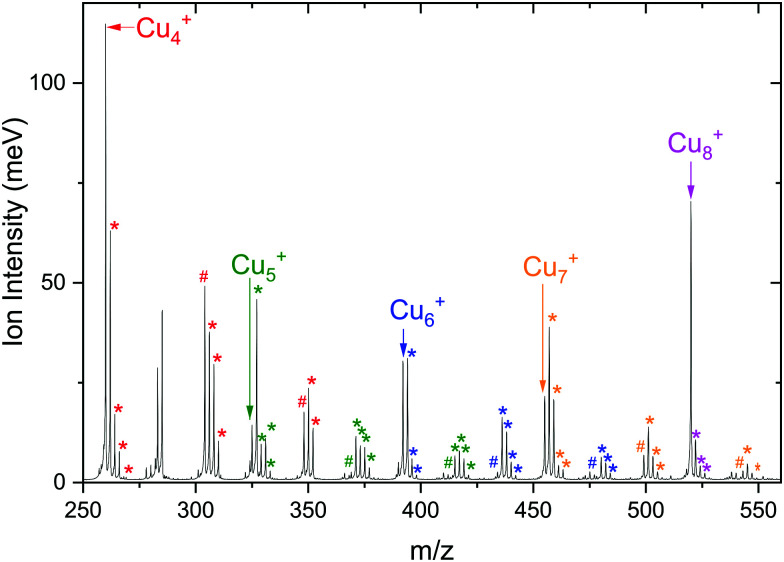
Mass spectrum of [Cu_*n*_, *m*CO_2_, *p*H_2_]^+^ with *n* = 4–8, *m* = 0–2, and *p* = 0–4 obtained using isotopically enriched ^65^Cu. Masses corresponding to complexes are color-coded according to the color of the bare cluster. The hash tag (#) indicates attachment of CO_2_ and the asterisk (*) of H_2_.

To see whether co-adsorption of H_2_ can activate CO_2_, we have examined the products formed upon reacting Cu_*n*_^+^ (*n* = 4–7) with a gas mixture of H_2_ and CO_2_. In the resulting mass spectrum, presented in Fig. 4, it is evident that apart from masses indicative for binding of the individual CO_2_ and H_2_ molecules, also co-adsorption complexes [Cu_*n*_, *m*CO_2_, *p*H_2_]^+^, typically with *m* = 1,2 and *p* = 1–4, are formed. The ion intensity of the complexes formed upon the adsorption of a single H_2_ molecule for *n* = 4 and 5 is much lower than that of the bare cluster, while for *n* = 6 and 7 it is the opposite. Complexes with *p* > 1 are always much lower in intensity than complexes with a single H_2_ adsorbed. For the sole adsorption of CO_2_, only the product [Cu_4_, CO_2_]^+^ is significantly higher in intensity than [Cu_4_, CO_2_, *p*H_2_]^+^. For other complexes, the intensity of just CO_2_ adsorbed on the cluster is lower than for the co-adsorption complexes: the intensity of [Cu_7_, CO_2_]^+^ is about half that of [Cu_7_, CO_2_, H_2_]^+^, while the intensities of [Cu_*n*_, CO_2_]^+^*n* = 5, 6 are almost negligible. The adsorption of a second CO_2_ leads to dominance of [Cu_*n*_, 2CO_2_, H_2_]^+^ for all *n*. Still, the ratio [Cu_4_, 2CO_2_]^+^ : [Cu_4_, 2CO_2_, H_2_]^+^ is significantly higher than the equivalent for other *n*. These patterns suggest that Cu_4_^+^ binds H_2_ more weakly than Cu_5–7_^+^, consistent with our earlier work.^50^

This is also expressed in the observed photofragmentation: the fragmentation pattern of co-adsorption complexes with Cu_4_^+^ suggests the sequential loss of H_2_ from [Cu_4_, 2CO_2_, *p*H_2_]^+^ until [Cu_4_, *m*CO_2_]^+^ is formed followed by the sequential loss of CO_2_, as illustrated in Scheme 1A. For Cu_*n*_^+^*n* = 5–7 a different fragmentation pattern was observed (Scheme 1B). The co-adsorption complexes first fragment *via* sequential loss of H_2_ to end as [Cu_*n*_, *m*CO_2_, H_2_]^+^. Then, rather than shedding the last H_2_, fragmentation continues by sequential CO_2_ loss to form [Cu_*n*_, H_2_]^+^. The bare cluster can be formed after the fragmentation of [Cu_*n*_, *p*H_2_]^+^ or [Cu_*n*_, *m*CO_2_]^+^. It is important to note that no loss of other fragments than H_2_ and CO_2_ has been observed. The difference in the fragmentation patterns suggests that the first H_2_ binds more strongly to Cu_5–7_^+^ than CO_2_. In contrast, CO_2_ binds more strongly to Cu_4_^+^ as also suggested by the intensity distribution of the measured mass channels.

### IR spectroscopy of [Cu_4_, CO_2_, H_2_]^+^

**Fig. 5:**
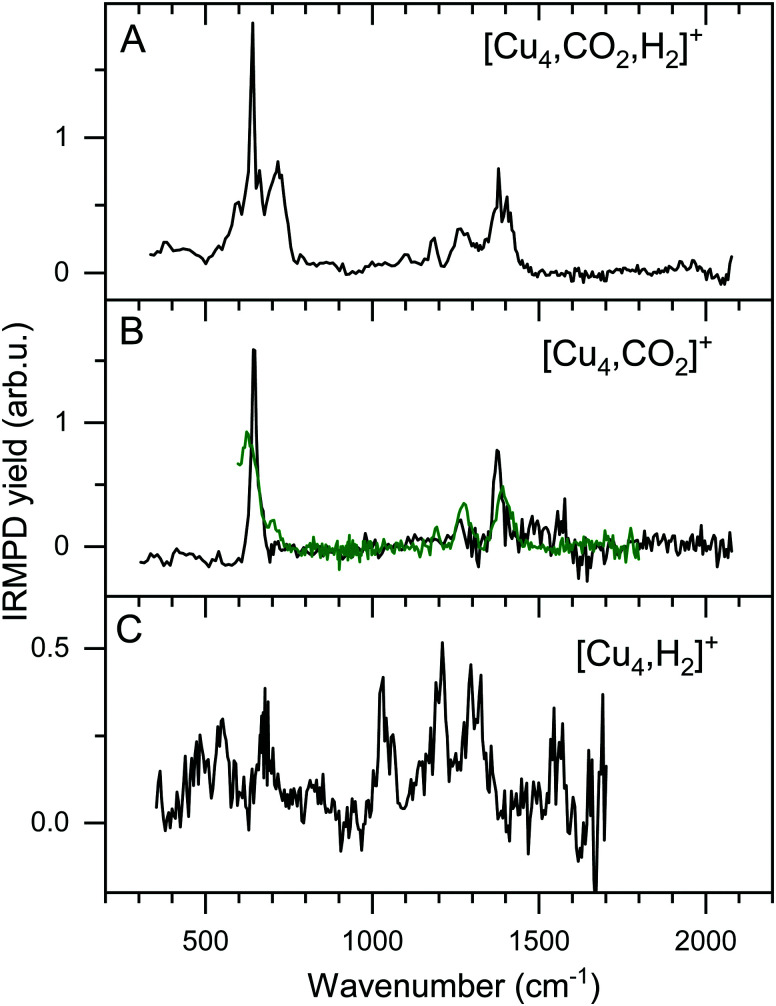
Experimental IRMPD spectra of [Cu_4_, CO_2_, H_2_]^+^ (A) and [Cu_4_, CO_2_]^+^ (B, black). Panel B also shows the [Cu_4_, CO_2_]^+^ reference spectrum from Fig. 3E (green trace), and Panel C that for [Cu_4_, H_2_]^+^ from ref. 50.

The focus of this work lies on reaction products where one H_2_ and one CO_2_ molecule are adsorbed on the cluster Cu_n_^+^. The obtained experimental spectrum for [Cu_4_, CO_2_, H_2_]^+^ is shown in Fig. 5 (top panel). This spectrum exhibits six bands at 641, 713, 1098, 1184, 1270, and 1391 cm^−1^. To interpret this spectrum, we compare it to experimental spectra of the cluster reacted with the individual molecules. Simultaneous with recording the spectrum for [Cu_4_, CO_2_, H_2_]^+^, we have obtained a spectrum for [Cu_4_, CO_2_]^+^. Note that this spectrum is reconstructed using the depletion of all fragments including higher-order complexes with multiple H_2_ and CO_2_ as illustrated in Scheme 1A. This method was applied in order to account for all possible ingrowth from [Cu_4_, *m*CO_2_, *p*H_2_]^+^ into this channel. The [Cu_4_, CO_2_]^+^ spectrum, shown in Fig. 5B (black trace), shows bands at 644, 1262, 1376, and 1573 cm^−1^. In green, the spectrum of [Cu_4_, CO_2_]^+^ from Fig. 3E is reproduced. In that experiment, no higher-order complexes were present and, therefore, this spectrum does not suffer from ingrowth. This spectrum of [Cu_4_, CO_2_]^+^ obtained during the co-adsorption experiment is fairly similar to that from Fig. 3E, but there are some minor differences. The spectrum in green exhibits two additional low-intensity bands. The band attributed to the CO_2_ bending vibration at 644 cm^−1^ gets a small shoulder at 704 cm^−1^ and the band at 1186 cm^−1^ probably originates from the antisymmetric stretch of CO_2_ probed with the second harmonic of FELICE. Similar bands were found in the data presented earlier in Fig. 2 for the Cu_*n*_^+^ (*n* = 7–25). The [Cu_4_, CO_2_]^+^ spectrum obtained from the co-adsorption experiments also has an additional feature at 1573 cm^−1^, but we interpret this as an artifact, just like the noisy (and in fact negative) signal just above 1600 cm^−1^. Therefore, the reference spectrum seems more reliable and we conclude that CO_2_ is bound to Cu_4_^+^ in end-on configuration, in the same manner as on Cu_*n*_^+^ (*n* = 7–25). If we compare spectra of the co-adsorbed complex [Cu_4_, CO_2_, H_2_]^+^ and the reference spectrum of [Cu_4_, CO_2_]^+^ it becomes clear that the bands at 641, 1184, 1270, and 1391 cm^−1^ in the [Cu_4_, CO_2_, H_2_]^+^ spectrum originate from intact CO_2_.

To interpret the remaining bands we turn to the spectrum of [Cu_4_, H_2_]^+^ we reported earlier,^50^ shown in Fig. 5C. This spectrum exhibits seven bands at 485, 551, 670, 1030, 1200, 1290 and 1555 cm^−1^. It is of interest to note that the spectrum of [Cu_4_, CO_2_, H_2_]^+^ was recorded with a lower laser power to prevent saturation of the strongest bands (now identified as originating from CO_2_ vibrations). Nevertheless, the remaining bands at 713 and 1098 cm^−1^ in the spectrum of [Cu_4_, CO_2_, H_2_]^+^, and the unresolved structure between 800 and 1050 cm^−1^, are similar to those observed for [Cu_4_, H_2_]^+^. The resemblance between the spectrum of [Cu_4_, H_2_]^+^ and that of the co-adsorption complex suggests that CO_2_ is weakly bound to the cluster, while H_2_ is molecularly or dissociative adsorbed, as found previously for [Cu_4_, H_2_]^+^.^50^

**Fig. 6:**
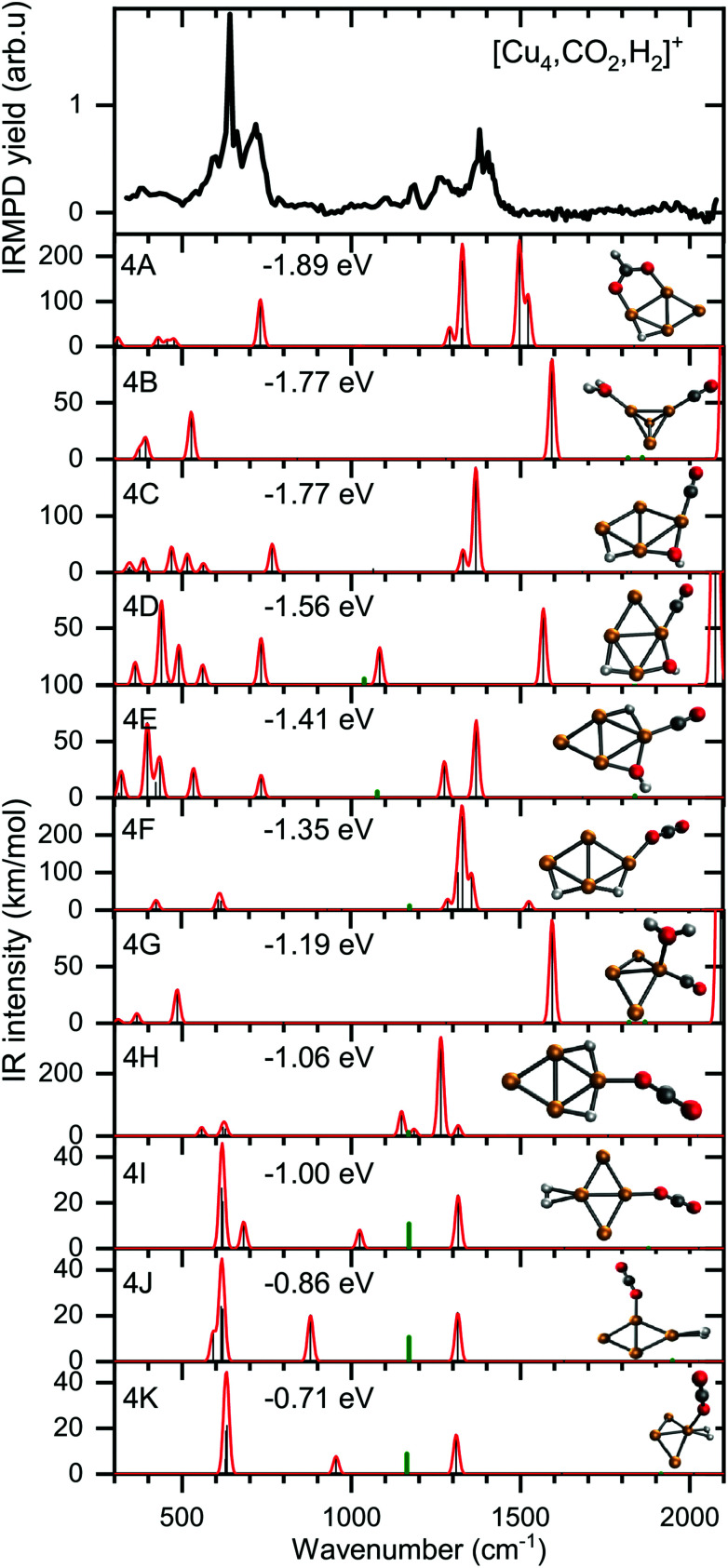
Experimental spectrum of [Cu_4_, CO_2_, H_2_]^+^ (top panel) compared to theoretical spectra of possible isomers. The calculated modes (black sticks) are complemented by a 20 cm^−1^ Gaussian convolution. The frequencies of calculated modes outside the spectral range probed (above 2100 cm^−1^) were divided by a factor of two and the intensities multiplied by 1% (green sticks) simulating the measurement with the second harmonic of FELICE. The energy relative to the free reactants of the corresponding structure is also shown.

To verify and rationalize this, we compare the experimental spectrum of [Cu_4_, CO_2_, H_2_]^+^ to calculated spectra of different isomers in Fig. 6. In the structure search, it was found that the majority of structures is based on the 2D rhombic geometry of the Cu_4_^+^ cluster, which was found to be present in the molecular beam in complexes with Ar and H_2_.^50,62^ The exception is formed by structure 4B, the second-lowest in energy, and the only one based on a pyramidal cluster structure. Low-energy structures are dominated by isomers where reduction of CO_2_ has led to the formation of water or hydroxyl and CO; structure 4A, the lowest energy structure found, has a formate on the cluster. Only the sixth-lowest energy isomer 4F has an intact CO_2_. The energy of formation of 4F (−1.35 eV with respect to the reactants Cu_4_^+^, H_2_, and CO_2_) is only 0.5 eV higher than that of 4A, suggesting CO_2_ reduction is thermodynamically favored, but not by very much.

When comparing the experimental spectrum with theoretical spectra, it is evident that among the lowest energy structures 4B–E and 4G cannot be the dominant species in the molecular beam. The spectra of these structures all exhibit quite strong bands below 600 cm^−1^ for which no evidence is found in the experimental spectrum. Of the remaining structures, 4A can also be ruled out since it exhibits strong bands above 1500 cm^−1^, while the highest frequency band on the experimental spectrum is at 1391 cm^−1^. The experimental band at 641 cm^−1^ could be assigned to strong bands at 638, 640, or 652 cm^−1^ from 4I, 4J, or 4K, respectively, or to the low-intensity bands at 634 (4F) and 647 cm^−1^ (4H). Structure 4J appears to be ruled out due to a strong band predicted at 910 cm^−1^, for which no experimental evidence is found. The band at 1391 cm^−1^ could be due to bands for 4F, 4H, 4I, and 4K (1372, 1307, 1361, and 1351 cm^−1^). A word of caution is in place here: these calculations are all done in the harmonic approximation, and inherently do not reproduce the CO_2_ Fermi resonance, which is likely still present for isomers 4F, 4H, 4I, and 4K, and it could thus also be seen back in the bump at 1270 cm^−1^. The experimental sideband of the strong 641 cm^−1^ resonance, centered at 713 cm^−1^, appears most plausibly assigned to 4I's band predicted at 703 cm^−1^. The 4I band at 1059 cm^−1^ could cause the experimental band at 1098 cm^−1^. We cannot rule out 4K despite its band at 986 cm^−1^, which could simply be too weak to detect. The experimental band at 1186 cm^−1^ could potentially originate from a FELICE second harmonic probing of the antisymmetric stretch of CO_2_ in isomers 4I and 4K, predicted at 2418 and 2404 cm^−1^, respectively.

All structures that could be responsible for the spectrum are formed by the reaction of rhombic Cu_4_^+^, which was found to be the most stable isomer,^62^ with CO_2_ and H_2_. In all cases, CO_2_ is bound to the cluster in a linear end-on configuration, as was seen earlier for bare Cu_*n*_^+^ clusters. The H_2_ is either molecularly bound to the obtuse apex of the cluster with CO_2_ bound to the same (structure 4K) or opposite Cu atom (4I), or dissociated on the obtuse apex and bound in-plane on bridge sites with CO_2_ bound to the acute apex for 4F. All these configurations of H_2_ are consistent with the previous work.^50^ The presence of structure 4H is difficult to rule out or confirm: its principal bands are all consistent with the structure above 1000 cm^−1^ in the experimental spectrum.

### Potential energy surface for the adsorption and activation reaction of CO_2_ and H_2_ on Cu_4_^+^ clusters

**Fig. 7:**
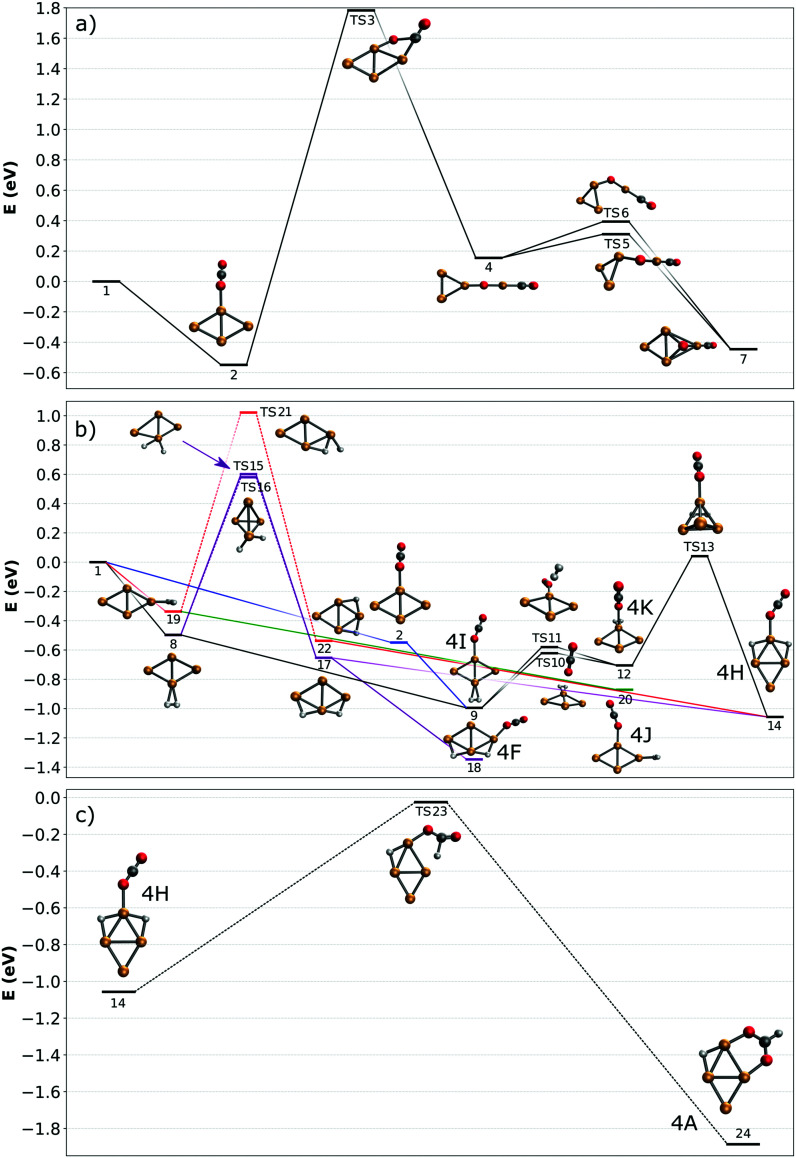
Potential energy surfaces describing adsorption reactions on Cu_4_^+^. (a) CO_2_ adsorption and dissociation; (b) co-adsorption of H_2_ and CO_2_ (color codes are used to identify different pathways in the text; dashed lines denote simplified pathways for which full path are shown in the ESI†); (c) CO_2_ reduction to formate. All energies are given with respect to the reactants.

To understand how the co-adsorption of CO_2_ and H_2_ proceeds and which products can be formed, the potential energy surface (PES) for this reaction is calculated and presented in Fig. 7, with the energies, ZPE corrected energies, enthalpies and Gibbs free energies in Table S1 (ESI†). The inclusion of the ZPE and the thermal effects in enthalpies play a relatively small role, while the entropy part is more important, especially when both CO_2_ and H_2_ are added to the cluster and the reactions progress. All structures included in the PES are numbered, the structures used for assignment of the [Cu_4_, CO_2_, H_2_]^+^ IRMPD spectrum in Fig. 6 are also labeled with the corresponding letters.

First, the adsorption and dissociation of CO_2_ over the bare cluster are evaluated in Fig. 7A. The entrance complex (2) is formed by linear CO_2_ adsorbed on the obtuse apex of rhombic Cu_4_^+^, with a CO_2_ binding energy of 0.54 eV. Adsorption on the acute apex (not shown) is only 0.08 eV higher in energy. Of all structures found, the entrance complex is thermodynamically the most favorable, followed by one (7, +0.09 eV) where CO_2_ is dissociated, with CO attached to the acute apex of the still close to rhombic cluster, and the loose O on a hollow site. Although both structures are exothermic with respect to the reactants, for CO_2_ to dissociate it has to overcome a barrier at +1.79 eV associated with transition state (TS3). The dissociation pathway involves a rearrangement of the cluster to allow the CO_2_ molecule to bend, leading to the insertion of a Cu atom into the C=O bond in structure (4). This barrier is obviously too high to be overcome, as is experimentally evidenced above.

In Fig. 7B, potential reaction paths for forming the co-adsorption complexes are explored. If the entrance complex (2) reacts with the H_2_, a co-adsorption complex 4I, with intact H_2_ adsorbed on the opposite obtuse apex of the cluster, can be formed barrierless as indicated by the blue route. This complex is more favorable than (2) by 0.46 eV. 4I can of course also be formed by first adsorbing H_2_, and then CO_2_ (route indicated in black). Entrance complex 4I can further rearrange to the slightly less favorable (0.29 eV) structure 4K, also an entrance complex, by transfer of either H_2_ or CO_2_ over the short axis of the cluster so that both molecules are bound on the same obtuse apex. Structure 4K can likely also be directly formed by adsorption of H_2_ onto structure (2), but it requires a bending of the CO_2_ ligand, which may not be energetically more favourable than the formation from structure 4I, given it is relatively low barriers. The H_2_ on 4K can dissociate by crossing transition state (TS13) at 0.74 eV with respect to 4K to form complex 4H. This reaction involves a compression–elongation-type interconversion of the horizontally oriented rhombus into a vertically oriented one, *via* a tetrahedron in TS13, again showing the cluster's dynamic nature. Although this barrier is the highest in this reaction path, it is only 0.04 eV higher than the energy of the reactants and might be overcome at room temperature. 4H has two H atoms on bridging sites, sharing the acute apex, with CO_2_ in end-on configuration in-between. This configuration is thermodynamically more favorable than 4I by 0.07 eV. There is an alternative pathway to form 4H, but it requires H_2_ dissociation before CO_2_ adsorption. In the current calculations, the barrier towards dissociation is at least 0.58 eV above the energy of the reactants, making it difficult to see this as a viable pathway. However, this barrier was at the PBE/QZV4P level calculated to be only 0.15 eV, making the reaction much more plausible. In fact, structure (22) was one of the assigned species for the spectrum of H_2_ adsorbed onto Cu_4_^+^.^50^ To investigate this somewhat better, we benchmarked this barrier for the two functionals PBE, TPSSh with various (larger) basis sets and with CCSD(T) (ESI†). We find that the level of correlation and relativistic effects each lead to an error of ∼0.2 eV in the computed relative energies, and that the barrier height is very sensitive to the structures. Given the earlier assignment of (22) to a H_2_ adsorption product of Cu_4_^+^,^50^ we assume that this route is open in the current experiment, too. The adsorption of CO_2_ onto structure (22) will directly lead to 4H, that onto structure 17 to 4F, at −1.35 eV the lowest energy structure in this PES.

To summarize: complexes 4H, 4I, 4K, and 4F, which are viable candidate structures for assignment of the experimental bands, can all be formed relatively easily. Each pathway leads to the formation of complexes with linear CO_2_ bound in end-on configuration. Structures 4I and K are formed most easily, requiring no H_2_ dissociation, whereas formation of structure 4H can proceed *via* several paths, which often requires the crossing of a relatively high, but not insurmountable, barrier. The energetically most feasible (black) route for 4H also allows the formation of structures 4I and 4K. Of these, the former is the most stable, and appears the most important candidate for assignment. The energetically most favorable structure, 4F, also requires dissociation (purple route).

The co-adsorption complexes discussed above (4F, I–K) could of course also lead to dissociation of CO_2_. However, if we assume that the corresponding barrier is similar to the one calculated for CO_2_ on bare Cu_4_^+^ (Fig. 7A), it can be expected that, even starting from the lowest energy structure 4F, the transition state lies around 1 eV higher than the reactants. Therefore, the energetic gain from the adsorption of H_2_ is not enough to overcome this barrier, and dissociation of CO_2_ will not be examined further.

In contrast, the barrier for CO_2_ reduction to formate, which is energetically also more favorable than CO_2_ dissociation, is almost isoenergetic with the reactants, making this route more plausible than dissociation. A reduction pathway starting from 4H is illustrated in Fig. 7C. Here, the CO_2_ ligand leans over to one of the bridging H atoms, abstracting it to form HCOO (formate), which then rotates along the Cu–O–C bond to bind *via* the second O atom in a η^2^ bidentate configuration to Cu_4_^+^. An additional minimum and transition state were located on the potential energy surface (see Fig. S11 in the ESI†), however the minimum is shallow and thus expected to play a negligible role in the reaction. Since many routes in Fig. 7B lead to the formation of 4H, it acts as a ‘gateway’ structure for the formation of formate. Although we can rule out CO_2_ reduction as the dominant pathway in the current experiments, the pathway calculated suggests it is not entirely out of reach. While the decreased entropy of the formate adduct compared to the free reactants has certainly an adverse effect, its Gibbs-free energy is still negative (see Table S1 in the ESI†), so its formation is thermodynamically allowed at 298 K. Thus, we can speculate that its formation under thermalized conditions in an ion trap only requires moderate temperatures. Because several computational studies of CO_2_ hydrogenation to methanol over Cu clusters suggest that the methanol formation proceeds *via* formate,^11,48,49^ this opens up the possibility to complete a full catalytic cycle over Cu_4_^+^.

### IR spectroscopy of [Cu_*n*_, CO_2_,H_2_]^+^ (*n* = 5–7)

**Fig. 8:**
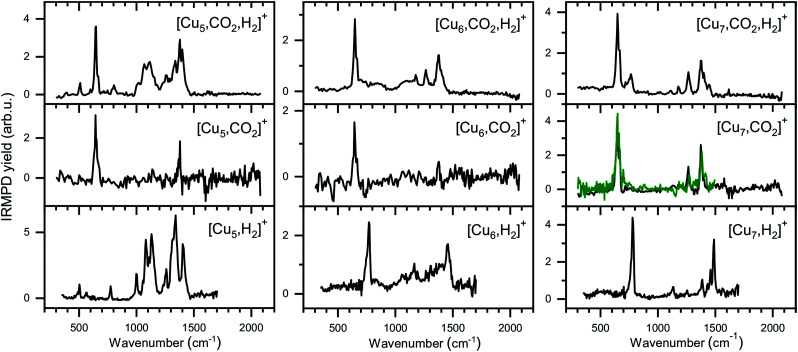
Experimental spectra of [Cu_*n*_, CO_2_, H_2_]^+^, [Cu_*n*_, CO_2_]^+^, and [Cu_*n*_, H_2_]^+^ for *n* = 5–7 presented in a similar manner as for the Cu_4_^+^ complexes in Fig. 6. The green trace on the [Cu_7_, CO_2_]^+^ spectrum is the reference spectrum from Fig. 3 obtained during individual CO_2_ adsorption experiments.

The experimental spectra for the co-adsorption of CO_2_ and H_2_ onto Cu_*n*_^+^ (*n* = 5–7) are presented in Fig. 8 (top panels), together with spectra for the individual adsorption of CO_2_ (middle) and H_2_ (bottom). The depletion yield spectrum of [Cu_5_, CO_2_]^+^ has been reconstructed from the same measurement, but in contrast to the spectrum for [Cu_4_, CO_2_]^+^, here only the signals of [Cu_5_, *m*C, 2*m*O]^+^ (*m =* 1,2) have been used, since complexes with H_2_ do not fragment into these mass channels (see Scheme 1B).

The spectrum of [Cu_5_, CO_2_, H_2_]^+^ shows ten bands at 511, 646, 802, 1008, 1068, 1118, 1260, 1335, 1384 and 1640 cm^−1^, where bands between 950 and 1500 cm^−1^ strongly overlap. Bands at 646 cm^−1^, 1260 and 1335 cm^−1^ can safely be assigned to the CO_2_ bending vibration and the Fermi dyad, even though the spectrum of [Cu_5_, CO_2_]^+^ only shows one of the Fermi dyad bands, with the second probably hidden in the relatively high noise level. The other bands are lower in intensity, but their overlap with the bands measured for [Cu_5_, H_2_]^+^ is recognizable: bands at 503, 775, 1002, 1081, 1135, 1260, and 1330 cm^−1^ for [Cu_5_, H_2_]^+^ appear to correspond to bands at 511, 802, 1008, 1068, 1118, and 1335 cm^−1^ in the spectrum of the co-adsorption product; other bands visible in the [Cu_5_, H_2_]^+^ spectrum are likely hidden in the shoulders of the strong CO_2_ bands. Interestingly, a new potential band is observed for the co-adsorption product around 1640 cm^−1^. This band is absent in the experimental spectra of the individual complexes or in the calculated spectra of complexes with H_2_. This might be indicative of a reaction between CO_2_ and H_2_ on the cluster surface, but we are unable to assign this band without calculated structures. Nevertheless, based on a comparison between experimental spectra alone, it is clear that the spectrum is dominated by the vibrations of both molecularly and dissociatively chemisorbed H_2_ together with CO_2_ adsorbed in the end-on configuration as it is found for Cu_4_^+^.

The case for Cu_6_^+^ is not much different. The experimental spectrum of [Cu_6_, CO_2_, H_2_]^+^ (Fig. 8, middle) exhibits seven bands at 647, 720, 860, 1086, 1175, 1264 and 1377 cm^−1^. Bands in the region between 700 and 1150 cm^−1^ are rather broad and, therefore, not so well defined. The quality of the spectrum of [Cu_6_, CO_2_]^+^ is not the greatest, but it also shows two bands at 646 and 1377 cm^−1^. Therefore, we interpret the bands at 647, 1264, and 1377 cm^−1^ in the [Cu_6_, CO_2_, H_2_]^+^ spectrum as vibrations of intact CO_2_. The remaining bands in the [Cu_6_, CO_2_, H_2_]^+^ spectrum exhibit a pattern quite similar to the spectrum of [Cu_6_, H_2_]^+^. The 720 cm^−1^ band has a rather broad structure (FWHM at least 90 cm^−1^), and forms a shoulder of the band at 647 cm^−1^ and its origin could be similar to the band observed for [Cu_6_, H_2_]^+^ at 770 cm^−1^. The weak band around 860 cm^−1^ observed for [Cu_6_, CO_2_, H_2_]^+^ can probably be linked to a shoulder of the 770 cm^−1^ bands in the [Cu_6_, H_2_]^+^ spectrum which peaks around 830 cm^−1^. Bands at 1067 and 1166 cm^−1^ in the [Cu_6_, H_2_]^+^ spectrum could correspond to the bands at 1086 and 1175 cm^−1^ on the [Cu_6_, CO_2_, H_2_]^+^ spectrum, respectively, while bands at 1306 and 1392 cm^−1^ for [Cu_6_, H_2_]^+^ could be hidden in the elevated baseline of overlapping bands. The exception is formed by the band at 1453 cm^−1^ in [Cu_6_, H_2_]^+^ spectrum, which is absent in the co-adsorption spectrum or shifted by more than 50 cm^−1^ forming the shoulder of an asymmetric band at 1377 cm^−1^. The origin of this band in the [Cu_6_, H_2_]^+^ spectrum is still unclear, and even after an extensive search no structure was found that could explain it.^50^ Nevertheless, we conclude that Cu_6_^+^ binds CO_2_ in end-on configuration with H_2_ co-adsorbed.

Finally, the spectrum for [Cu_7_, CO_2_, H_2_]^+^ is compared to its counterparts in Fig. 8 (right panels). The spectrum of the co-adsorption product is better resolved than those for [Cu_*n*_, CO_2_, H_2_]^+^ (*n* = 4–6), showing bands at 646, 766, 1117, 1182, 1264 and 1377 cm^−1^. The band at 1377 cm^−1^ has shoulders peaking at 1402 and 1444 cm^−1^. The spectrum for [Cu_7_, CO_2_]^+^ is in good agreement with the spectrum presented in Fig. 2, reproduced as a green trace in Fig. 8, and shows bands that are probably the same as the band 646, 1264 and 1377 cm^−1^ observed for [Cu_7_, CO_2_, H_2_]^+^. The strongest band for [Cu_7_, H_2_]^+^ at 784 cm^−1^, can be linked to the 766 cm^−1^ band for [Cu_7_, CO_2_, H_2_]^+^, slightly shifted but still clearly visible. The same is valid for the 1135 and 1488 cm^−1^ bands of [Cu_7_, H_2_]^+^, which correspond to a band at 1117 cm^−1^ and a shoulder of the 1377 cm^−1^ band for [Cu_7_, CO_2_, H_2_]^+^. The [Cu_7_, H_2_]^+^ bands at 1255 and 1386 cm^−1^ lie fairly close to the Fermi dyad of CO_2_ and therefore probably overlap. The only unexplained low-intensity bands in the [Cu_7_, CO_2_, H_2_]^+^ spectrum are found at 1182 and potentially at 1622 cm^−1^. We speculate that the band at 1182 cm^−1^ is caused by the antisymmetric stretch of CO_2_ excited by the second harmonic of FELICE (found at 1185 cm^−1^ in Fig. 3). We have no clear explanation for the 1622 cm^−1^ band, but due to its weakness, we do not regard this as crucial. We conclude again that CO_2_ binds only weakly to Cu_*n*_^+^ clusters (*n* = 4–7), even when H_2_ is co-adsorbed.

## Conclusion

We have recorded experimental IRMPD spectra for the products resulting from reacting Cu_n_^+^ (*n* = 4–7) clusters with CO_2_, and with CO_2_ and H_2_ simultaneously. The spectra of the clusters with only CO_2_ adsorbed are indicative for simple physisorption of the CO_2_ molecule in an end-on configuration, leaving the CO_2_ fundamental vibrations largely unchanged. This is in agreement with DFT calculations, which predict the activation of CO_2_ by cationic clusters is hindered by a barrier of at least 2.33 eV relative to the energies of the reactants. This inactivity of Cu_*n*_^+^ cations towards CO_2_ is also consistent with previous calculations, suggesting that CO_2_ only gets activated by a significant amount of charge transfer that goes hand in hand with stronger bonding.

When both H_2_ and CO_2_ co-adsorb onto the clusters, CO_2_ activation is not achieved either. No size-dependent effects have been observed in the binding of CO_2_ to either bare and H_2_ preloaded clusters. On the other hand, co-adsorption of CO_2_ does not affect the cluster size dependence of H_2_ adsorption as identified in ref. 50. DFT calculations of the reaction pathway for CO_2_ reduction over Cu_4_^+^ in which dissociative adsorption of H_2_ leads to H(a) being formed that could react with CO_2_ after migration from its absorption site on the cluster, thereby following a Langmuir–Hinshelwood type mechanism. The transition states along this reaction path are only slightly higher than the energy of the reactants, but the barriers might be in the order of 1 eV. This suggests that, although CO_2_ reduction under the current experimental conditions may not be feasible or dominant, it could be observed at only slightly higher temperatures and longer reaction times that can be provided by an ion trap. If such experiments would be successful, a study of the efficiency of CO_2_ reduction on more complex cluster materials could provide an understanding of *e.g.*, the promotor materials in the real catalyst.

## Conflicts of interest

There are no conflicts to declare.

## Supplementary Material

CP-023-D1CP03119H-s001
