# Supplementary material for: IR spectroscopic characterization of the co-adsorption of CO2 and H2 onto cationic Cun+ clusters
Source: Phys Chem Chem Phys. 2021 Oct 28;23(47):26661–73. doi: 10.1039/d1cp03119h (PMC8653698; doi:10.1039/d1cp03119h)
Supplement: CP-023-D1CP03119H-s001 [file CP-023-D1CP03119H-s001.pdf]

Electronic Supplementary Material (ESI) for Physical Chemistry Chemical Physics.
This journal is © the Owner Societies 2021

IR spectroscopic characterization of the co-adsorption of CO_2 and H_2 onto cationic Cu_n^+ clusters

Olga V. Lushchikova,[a] Máté Szalay,[b] Hossein Tahmasbi,[c] Ludo Juurlink,[c] Jörg Meyer,[c] Tibor Höltzl,[b,d] and Joost M. Bakker[*a]

Radboud University, Institute for Molecules and Materials, FELIX Laboratory, Toernooiveld 7, 6525 ED Nijmegen, The Netherlands.
MTA-BME Computation Driven Chemistry Research Group, Department of Inorganic and Analytical Chemistry, Budapest University of Technology and Economics, Muegyetem rkp. 3, Budapest 1111, Hungary.
Leiden Institute of Chemistry, Gorlaeus Laboratories, Leiden University, P.O. Box 9502, 2300 RA Leiden, The Netherlands
Furukawa Electric Institute of Technology, Késmárk utca 28/A 1158. Budapest, Hungary

Supporting Information

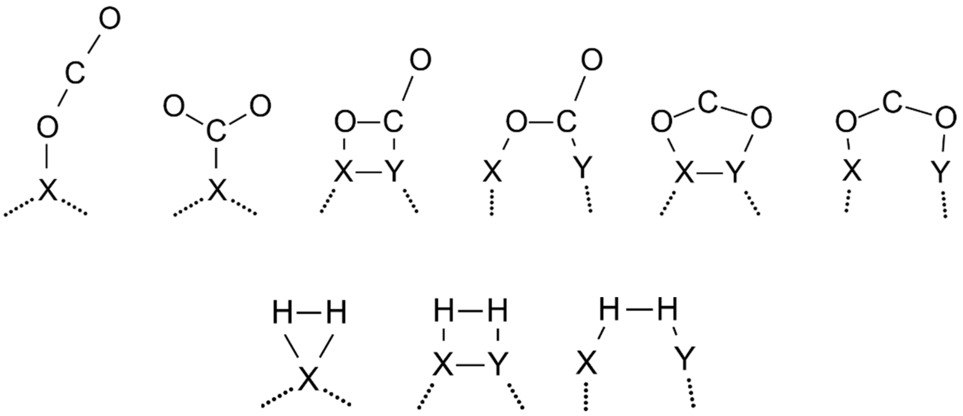

Figure S1: Different adsorbate binding modes. X, Y represent the cluster atom. Continuous lines indicate the adsorbate binding mode, while dashed line indicate that the atoms X and Y belong to the cluster (not the actual coordination number). See the text for the notations.

The initial geometries for the cluster-adsorbate systems were systematically generated using our program based on the Molmod library.[1] We generated possible structures as follows:

- Rhombic Cu_4^+ + CO_2 (X, Y=Cu)
- Rhombic Cu_4^+ + CO + O (X=Cu, O, Y=Cu, C, O)
- Tetrahedral Cu_4^+ + CO_2 (X, Y=Cu)
- Tetrahedral Cu_4^+ + CO + O (X=Cu, O, Y=Cu, C, O)
- Rhombic Cu_4^+ + H_2 (X=Cu, Y=Cu)
- The most stable intact H_2 isomer of $[Cu_{4,}H_2]^+$ + CO_2 (X = Cu, Y=Cu, O, C)
- The most stable dissociated H_2 isomer of $[Cu_{4,}H_2]^+$ + CO_2 (X = Cu, Y=Cu)
- The most stable dissociated H_2 isomer of $[Cu_{4,}H_2]^+$ + CO + O (X=Cu, Y=Cu, C, O)
- The most stable Cu_4^+COO + H_2 (X = Cu, Y=Cu, O, C)

[1] Verstraelen, T: Molmod software Library, https://molmod.ugent.be/software

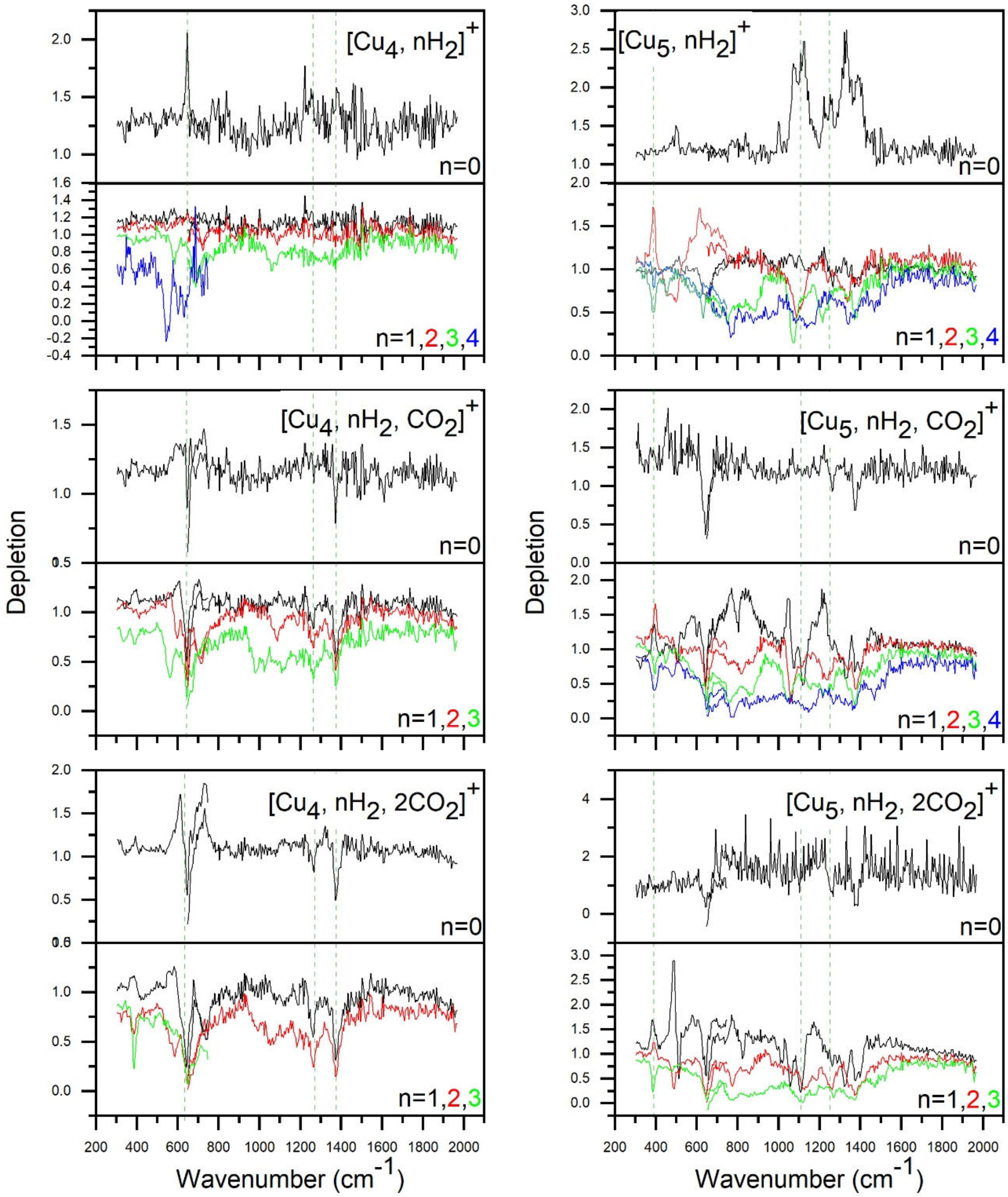

Figure S2: Raw depletion spectra for $[Cu_4,mCO_2,nH_2]^+$ and $[Cu_5,mCO_2,nH_2]^+$ with *n*=0-3, and *m*=0-2. Values exceeding 1 indicate a growth of the mass channel intensity, values below 1 indicate a reduction.

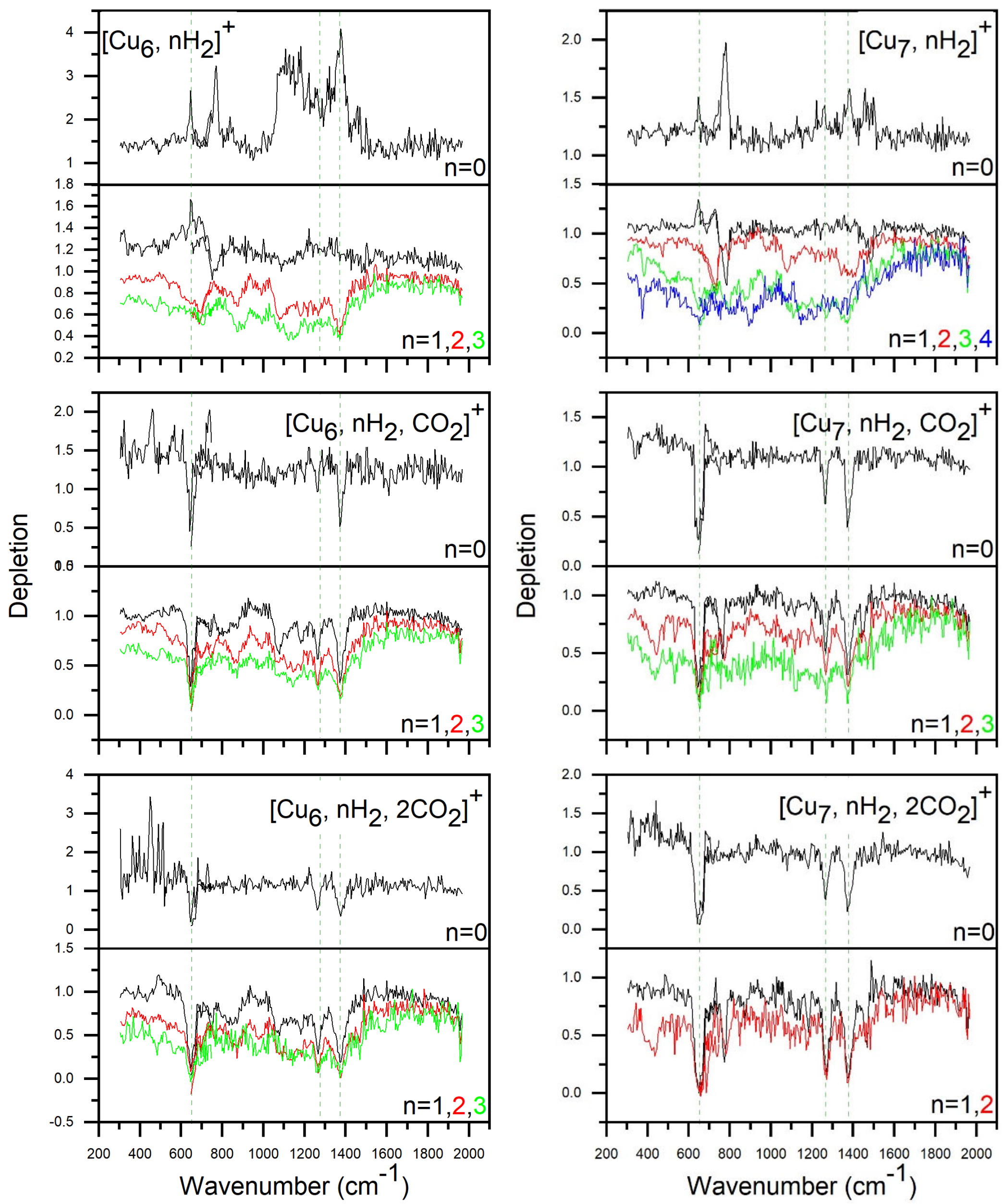

Figure S3: Raw depletion spectra for $[Cu_6,mCO_2,nH_2]^+$ and $[Cu_7,mCO_2,nH_2]^+$ with *n*=0-3, and *m*=0-2.

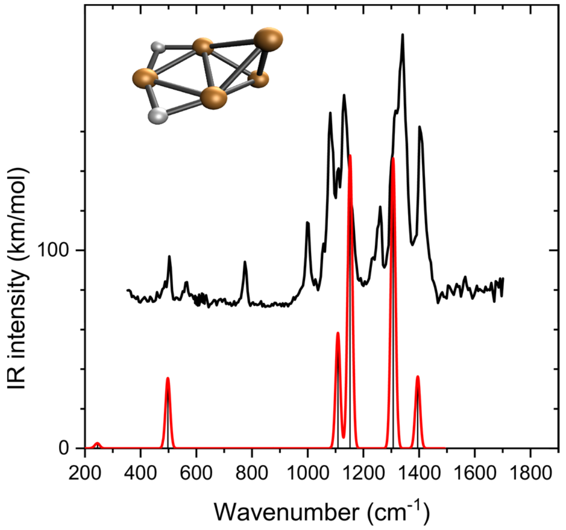

Figure S4: Experimental IRMPD spectrum of $[Cu_5,H_2]^+$ from Ref [51] and the harmonic spectrum of the structure indicated calculated at TPSSh/def2-TZVP + D3 level of theory. The harmonic frequencies are scaled by a factor of 0.968.

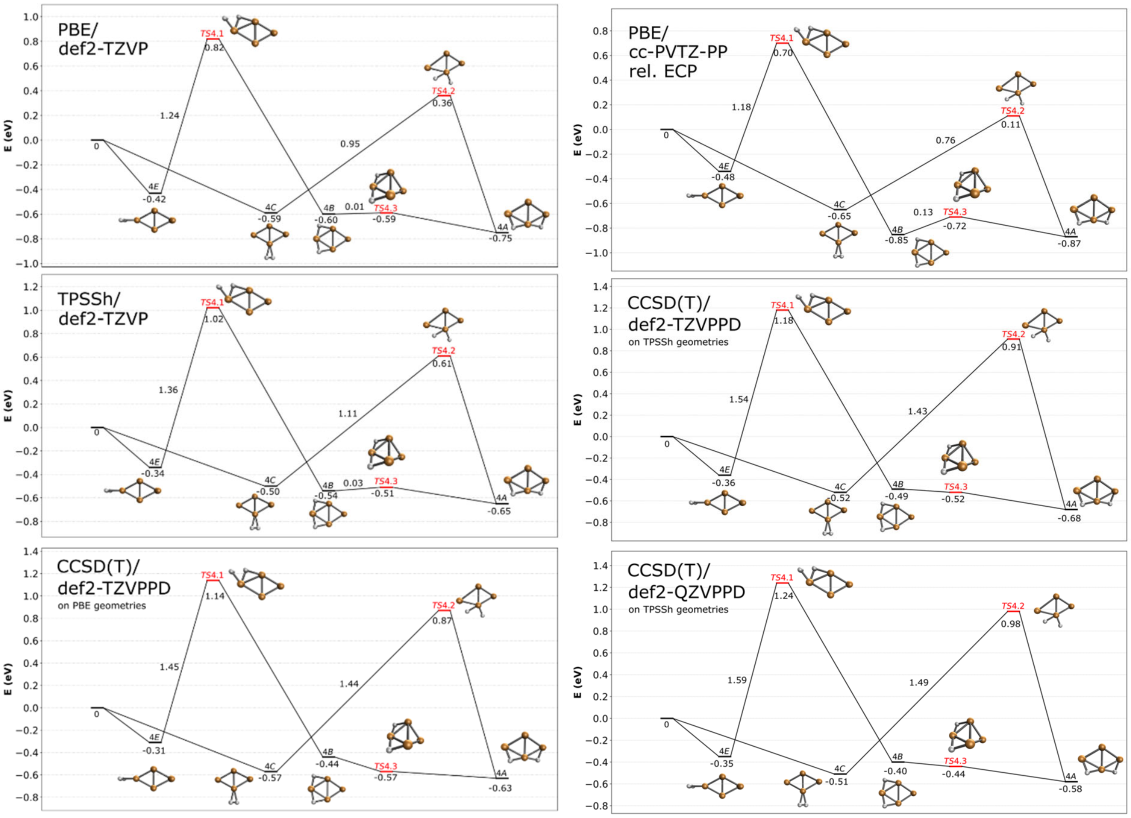

Figure S5: H_2 dissociation reactions paths on Cu_4^+, computed using different DFT functionals (PBE – GGA, and TPSSh – hybrid meta GGA) and CCSD(T), and different basis sets. The importance of relativistic effects was estimated from the comparison of the PBE results with the def2-TZVP all-electron and the cc-pVTZ-PP basis set with the relativistic Effective Core Potential.

The CCSD(T) single point computations on the DFT geometries place TS4.3 lower in energy than 4B. We believe that due to the very flat potential energy surface, the accurate structure of the optimum is important. In case of these CCSD(T) calculations optimization is not feasible due to the computational cost; therefore, the reaction energies can vary to some extent, depending on which the method was used to optimize the geometries. The energies of the CCSD(T)/def2-TZVPPD calculation on PBE or TPSSh geometries differ by 0.05 eV, and the barrier between 4B and 4A structures is only 0.01-0.04 eV depending on the method. This can explain why in the case of CCSD(T) calculations the TS4.3 are lower in energy than 4B.

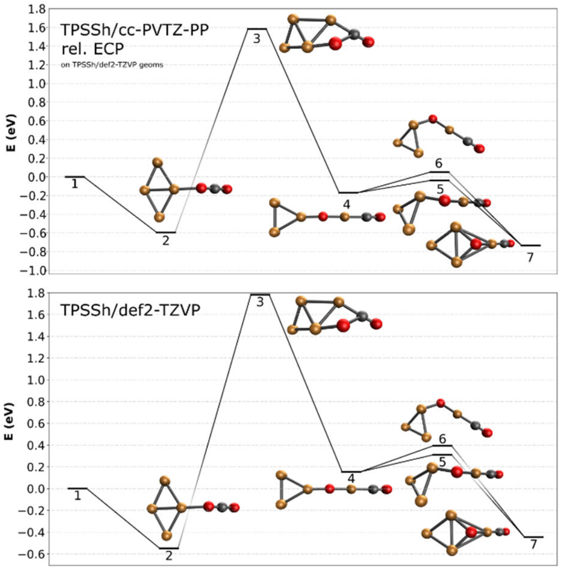

Figure S6: Comparison of the basis sets cc-PVTZ-PP with relativistic ECP and def2-TZVP for the CO_2 dissociation PES over Cu_4^+. The average difference between the reaction energies is 0.2 eV.

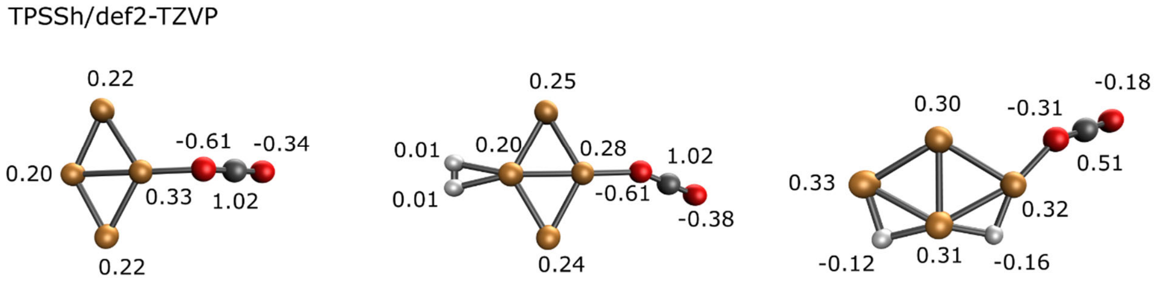

Figure S7: Natural charges of the Cu_4^+ adducts, using NBO calculations with TPPSh/def2-TZVP

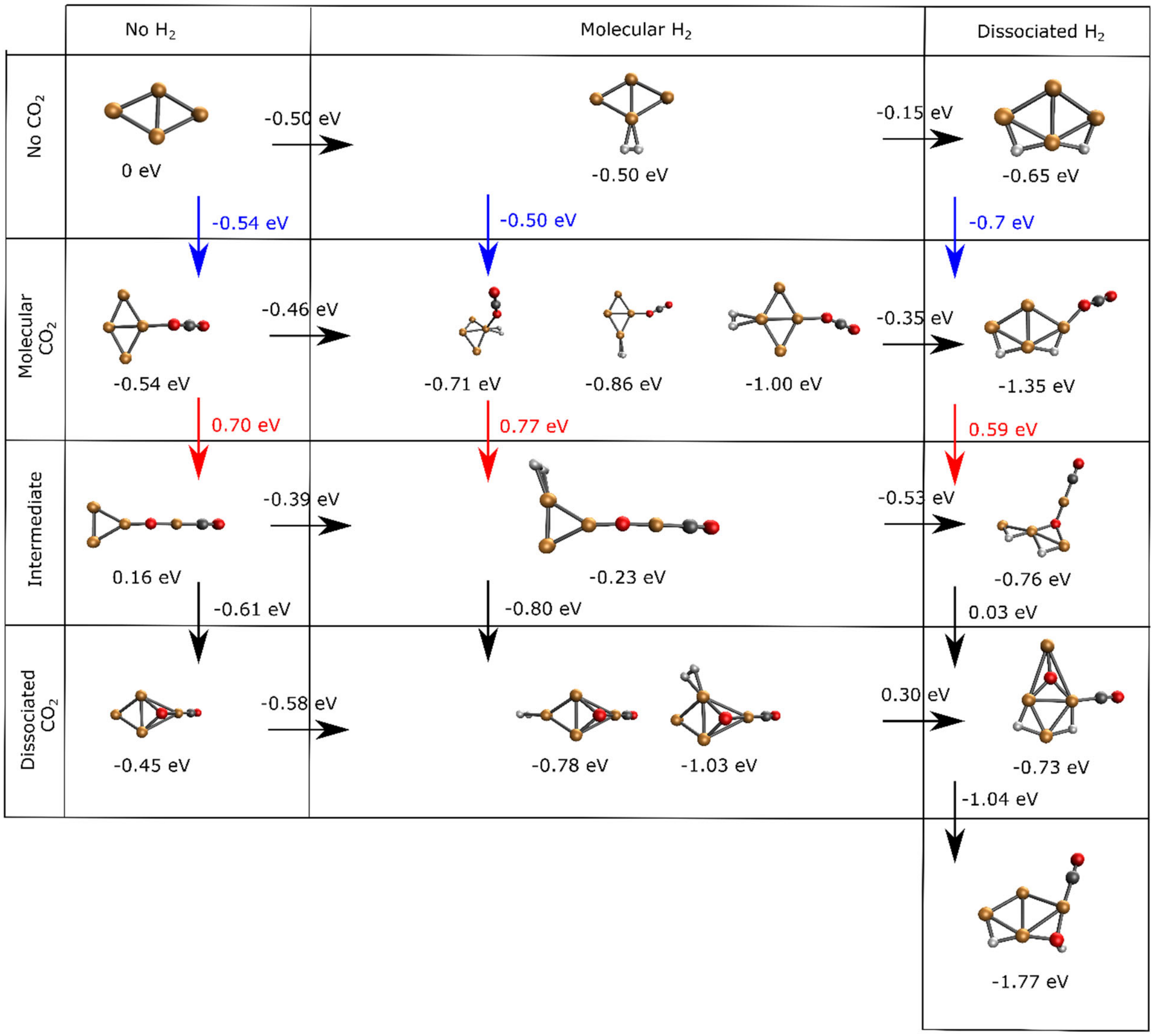

Figure S8: The proposed intermediates of the dissociation of CO_2 on bare Cu_4^+, Cu_4^+ with molecularly adsorbed H_2, and Cu_4^+ with dissociated H_2. The arrows show the energy differences between the geometries shown.

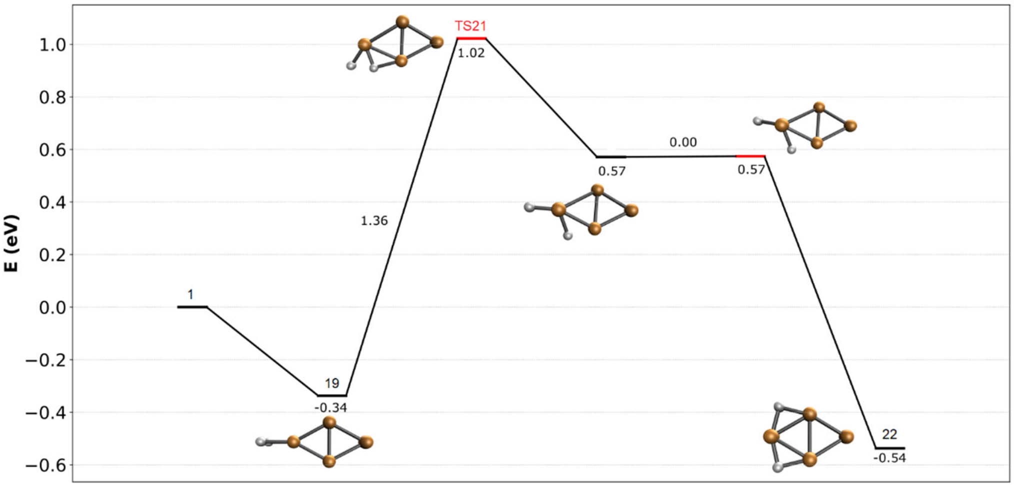

Figure S9: Detailed view of the dissociation pathway of H_2 on Cu_4^+, connecting structures 19 and 22 (red path) in Figure 7b.

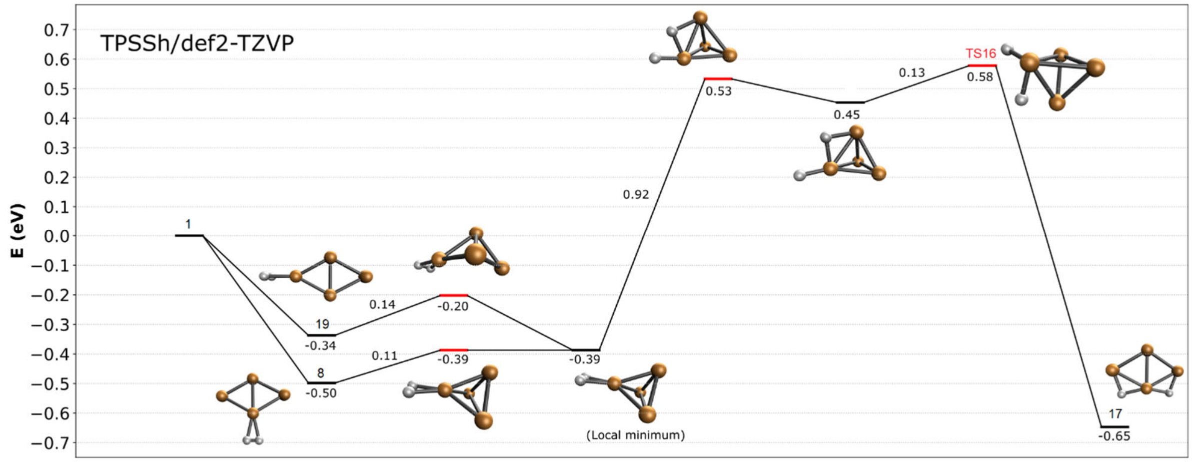

Figure S10: Detailed view of the dissociation pathway of H_2 on Cu_4^+, connecting structures 8 and 17 (violet path) in Figure 7b.

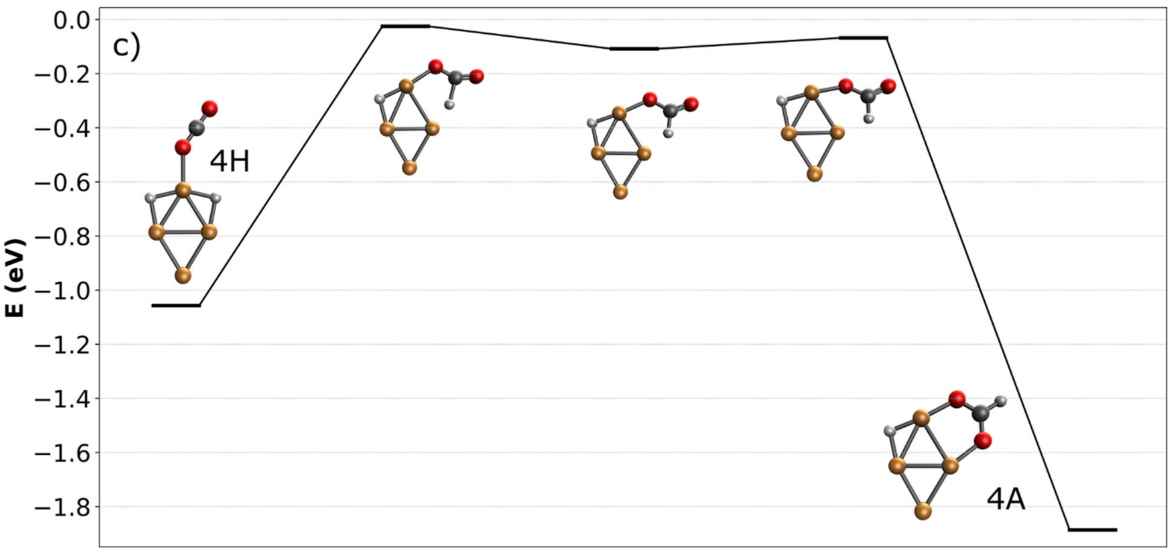

Figure S11: Detailed view of the CO_2 reduction pathway on Cu_4^+, connecting structures 14 (4H) and 24 (4A) in Figure 7c.

Table S1: Relative energies (E), zero-point energy corrected relative energies (E+ZPE) enthalpies (H) and Gibbs-free energies (G) with respect to the reactants of the structures described in the PES (Figure 7).The thermal corrections are computed at 298K and 1bar.

	E [eV]	(E+ZPE) [eV]	H [eV]	G [eV]
1	0.00	0.00	0.00	0.00
2	-0.54	-0.52	-0.50	-0.23
TS3	1.79	1.72	1.72	2.12
4	0.16	0.12	0.12	0.54
TS5	0.31	0.27	0.26	0.67
TS6	0.40	0.37	0.36	0.75
7	-0.44	-0.46	-0.46	-0.06
8	-0.50	-0.38	-0.42	-0.11
9	-0.99	-0.85	-0.88	-0.29
TS10	-0.62	-0.50	-0.54	0.04
TS11	-0.59	-0.53	-0.52	-0.06
12	-0.71	-0.59	-0.60	-0.03
TS13	0.04	0.04	-0.01	0.70
14	-1.06	-0.93	-0.99	-0.30
TS15	0.61	0.64	0.57	0.93
TS16	0.59	0.62	0.55	0.95
17	-0.65	-0.55	-0.61	-0.26
18	-1.35	-1.20	-1.25	-0.59
19	-0.34	-0.25	-0.27	0.00
20	-0.87	-0.76	-0.79	-0.18
TS21	1.02	1.01	0.95	1.31
22	-0.54	-0.44	-0.51	-0.16
TS23	-0.03	0.08	0.00	0.82
24	-1.89	-1.60	-1.69	-0.82

Table S2: XYZ coordinates of the different structures.

#	XYZ Coordinates				#	XYZ Coordinates			
Cu_4	Cu	-0.00004	-1.16979	-0.00058	CO_2	C	0.00000	0.00000	0.00000
	Cu	-2.12004	-0.00007	0.00058		O	0.00000	0.00000	1.16338
	Cu	0.00002	1.16996	-0.00058		O	0.00000	0.00000	-1.16338
	Cu	2.12005	-0.00010	0.00058					
H_2	H	0.00000	0.00000	-0.37133	2	Cu	0.50205	0.02696	0.10907
	H	0.00000	0.00000	0.37133		Cu	-0.79079	2.09126	0.02200
						Cu	-1.83309	-0.05296	-0.14140
						Cu	-0.64432	-2.12001	0.02923
						O	4.75416	0.03598	-0.36287
						C	3.65393	0.07101	-0.03232
						O	2.53266	0.10921	0.31860
TS3	Cu	1.18563	-1.25700	0.13501	4	Cu	2.67983	1.14821	-0.00643
	Cu	-1.51412	-0.86171	-0.11704		Cu	-2.96317	0.01076	0.00460
	Cu	0.13981	0.84940	0.44337		Cu	0.58071	0.01406	0.01136
	Cu	2.37097	0.71182	-0.41298		Cu	2.65065	-1.17183	-0.00581
	O	-1.68844	0.83709	0.75221		O	-1.18265	0.03766	0.01550
	C	-3.03148	0.30867	-0.42186		C	-4.78736	-0.01451	-0.01099
	O	-3.94874	0.95232	-0.61109		O	-5.91342	-0.03111	-0.02074
TS5	O	0.88662	-0.28121	0.93936	TS6	Cu	0.65219	-1.26595	-0.24962
	O	5.14906	0.23968	-1.04866		Cu	-2.08819	0.50173	0.14650
	Cu	-0.65810	0.55340	0.76881		Cu	0.86740	1.08022	-0.34359
	Cu	2.47965	-0.06680	0.15995		Cu	2.71551	-0.28120	0.34683
	Cu	-1.73786	-1.32351	-0.17801		O	-0.70221	1.61177	0.24383
	Cu	-2.60289	0.82366	-0.59939		C	-3.53580	-0.60070	0.08965
	C	4.12853	0.11941	-0.58580		O	-4.42850	-1.28739	0.05097
7	Cu	0.83083	-1.22757	0.38360	8	Cu	0.00042	-1.20979	-0.01895
	Cu	-2.04795	0.00206	0.16085		Cu	-2.11033	-0.06695	0.01734
	Cu	0.84257	1.23568	0.36913		Cu	2.11134	-0.06498	0.01727
	Cu	2.56437	-0.01092	-0.77362		Cu	-0.00024	1.14351	-0.01407
	O	-0.42933	0.01292	0.96416		H	-0.40773	2.86990	-0.02652
	C	-3.70281	-0.00390	-0.57776		H	0.37321	2.87804	-0.01943
	O	-4.73165	-0.00728	-1.03817					
9	Cu	0.525657	0.164314	0.024882	TS10	O	4.94724	0.00449	-0.05088
	Cu	-1.036285	2.005350	-0.006241		O	2.62092	0.03405	-0.02036
	Cu	-1.797195	-0.273749	-0.013923		H	-0.60049	0.36584	-2.88037
	Cu	-0.251694	-2.115144	0.001556		H	-0.57892	-0.41432	-2.87006
	O	4.827934	0.117415	-0.067331		Cu	-0.44553	-0.00109	1.20088
	C	3.701279	0.339483	-0.005827		Cu	-0.90858	2.06733	0.08479
	O	2.551914	0.575462	0.058186		Cu	-0.59608	-0.00250	-1.14758
	H	-3.586739	-0.229633	-0.048192		Cu	-0.88110	-2.07670	0.08722
	H	-3.433702	-0.992627	-0.025658		C	3.79030	0.01930	-0.03558
TS11	O	-4.76560	-0.07561	0.35234	12	Cu	-0.260532	-0.033301	-0.862837
	O	-2.53937	-0.22011	-0.30251		Cu	0.995192	-1.990529	-0.136977
	H	1.27965	0.10148	-3.46243		Cu	1.086728	0.060459	1.078346
	H	1.16004	0.26104	-2.74598		Cu	0.750701	2.071038	-0.162405
	Cu	-0.51923	-0.06010	-0.05709		O	-4.143684	-0.160707	1.177287
	Cu	0.85898	-2.06859	-0.02645		C	-3.338527	-0.133314	0.352941
	Cu	1.79408	0.10545	0.30663		O	-2.518418	-0.104801	-0.485801
	Cu	0.55509	2.12232	-0.02998		H	-0.671430	0.285385	-2.616318
	C	-3.66313	-0.14500	0.03494		H	-0.591146	-0.483803	-2.600936
TS13	O	-4.91926	0.04291	0.02966	14	Cu	-0.67075	-1.24014	-0.00011
	O	-2.63669	-0.07917	-0.39387		Cu	-2.86750	-0.22235	0.00016
	H	-0.11291	-0.60059	1.69491		Cu	-0.95730	1.26717	0.00003
	H	-0.15011	0.65914	1.67272		Cu	1.18900	0.23267	-0.00018
	Cu	-0.72315	-0.01681	0.24934		H	0.64119	1.75676	-0.00016
	Cu	1.20033	1.29352	0.58443		H	1.00288	-1.37914	-0.00019
	Cu	1.15455	-0.01215	-1.43840		C	4.28882	-0.07782	0.00015

	Cu	1.24577	-1.25333	0.62368		O	3.25446	0.48160	-0.00011
	C	-3.78946	-0.01571	-0.16780		O	5.30966	-0.60587	0.00041
TS15	Cu	2.09687	0.26751	-0.00004	TS16	Cu	0.07197	-1.45831	0.47356
	Cu	-0.13421	0.99032	-0.00002		Cu	-1.30528	0.11780	-0.75254
	Cu	0.36266	-1.33618	0.00005		Cu	-0.05738	0.96029	1.03046
	Cu	-2.27202	-0.05714	-0.00003		Cu	1.29821	0.25150	-0.73829
	H	0.12591	2.47200	0.00093		H	0.53922	1.89637	-0.19565
	H	-1.67154	1.45731	0.00026		H	-0.75706	1.83637	-0.18672
17	Cu	-0.00028	-1.30060	0.00340	18	Cu	0.478302	-1.178608	0.104729
	Cu	-2.11655	0.01057	-0.00338		Cu	-1.301023	0.673982	0.079482
	Cu	0.00006	1.17497	0.00332		Cu	1.097075	1.202191	-0.014284
	Cu	2.11675	0.01037	-0.00374		Cu	2.806116	-0.482677	-0.104405
	H	1.58005	1.51773	0.01282		H	2.714042	1.124915	-0.124293
	H	-1.57950	1.51837	-0.00128		H	-0.334328	1.915385	0.084059
						O	-5.310650	-0.519260	-0.252776
						C	-4.180041	-0.414136	-0.087938
						O	-3.018486	-0.329146	0.086241
19	Cu	0.06976	-1.15652	0.00376	20	Cu	0.489404	0.135222	0.072285
	Cu	-2.07811	0.00151	-0.00177		Cu	-1.260638	1.864569	0.007654
	Cu	0.06976	1.15611	0.00302		Cu	-1.738888	-0.488764	-0.109744
	Cu	2.20635	-0.00018	-0.00386		Cu	-0.094284	-2.234371	0.042624
	H	-3.87904	-0.04520	-0.40022		O	4.720771	0.533115	-0.241781
	H	-3.88595	0.01852	0.36702		C	3.594372	0.620387	-0.028099
						O	2.445319	0.721781	0.198214
						H	-1.762615	3.575516	0.448297
						H	-1.604578	3.639955	-0.302914
TS21	H	1.82276	-1.39837	0.00022	22	Cu	-0.00801	-1.29683	0.00001
	H	3.18649	-1.10518	-0.00859		Cu	-2.06381	0.00000	-0.00001
	Cu	0.06334	-1.10090	0.00211		Cu	-0.00801	1.29683	0.00001
	Cu	2.15581	0.10838	-0.00143		Cu	1.96508	-0.00000	-0.00001
	Cu	-0.21535	1.22047	0.00155		H	1.66386	1.57622	-0.00001
	Cu	-2.17654	-0.14162	-0.00194		H	1.66385	-1.57622	-0.00001
TS23	Cu	-0.11437	-0.98649	-0.00024	24	Cu	-0.03757	-0.95989	-0.00003
	Cu	-2.50561	-0.77116	0.00025		Cu	-2.41030	-0.60367	0.00004
	Cu	-1.14919	1.16912	-0.00017		Cu	-0.96588	1.27836	-0.00003
	Cu	1.32949	1.23173	0.00006		Cu	1.47067	1.10330	0.00002
	H	0.10874	2.19675	-0.00036		H	0.37658	2.21337	0.00002
	H	1.46117	-1.28465	-0.00045		H	3.61142	-1.97256	-0.00014
	C	2.91873	-0.96904	0.00008		C	2.72222	-1.33646	0.00008
	O	2.96094	0.26320	0.00051		O	2.90646	-0.08614	0.00002
	O	3.49759	-1.98205	-0.00012		O	1.59707	-1.90722	-0.00004
